# Docosahexaenoic acid intake and health in adults and older adults: a narrative review of disparities by country income level

**DOI:** 10.3389/fnut.2026.1742942

**Published:** 2026-03-20

**Authors:** Brenda Valle-Valdez, Xochitl Terrazas-Lopez, Alejandra Gonzalez-Rocha, Humberto Astiazaran-Garcia, Brianda Armenta-Guirado

**Affiliations:** 1 Department of Health Sciences, University of Sonora, Hermosillo, Mexico; 2 Social Interventions Research and Evaluation Network, University of California, San Francisco, San Francisco, CA, United States; 3 Department of Chemical and Biological Sciences, University of Sonora, Hermosillo, Mexico

**Keywords:** adults, older adults, docosahexaenoic acid (DHA), supplementation, high income countries, low-and middle-income countries, gross national income (GNI), health

## Abstract

Docosahexaenoic acid (DHA) is a long-chain omega-3 polyunsaturated fatty acid essential for maintaining optimal brain and cardiometabolic health across adulthood and aging. Despite its biological relevance, global evidence indicates substantial variability in DHA intake and status, largely influenced by dietary patterns, food availability, and socioeconomic conditions. This narrative review synthesized observational and interventional studies published between 2014 and 2026 that evaluated DHA intake or biomarkers in adults and older adults, emphasizing disparities according to country income level. Studies were categorized following the World Bank Gross National Income (GNI) classification and analyzed to describe intake patterns, biochemical concentrations, and their reported health associations. Important inequities were observed between countries. Populations in high-income countries (HICs) generally reported higher DHA intake and tissue concentrations, mainly due to regular fish consumption and greater access to supplementation, while evidence from middle-income countries (MICs) was scarce, heterogeneous, and based on small non-representative samples. In HICs, even moderate fish intake significantly improved DHA status, whereas in MICs, mean intakes frequently fell below 200 mg/day, a threshold commonly associated with cardiometabolic and neurocognitive benefits. Higher DHA levels were consistently linked to more favorable lipid profiles, lower triglyceride concentrations, and better cardiovascular indicators, though associations with blood pressure and mortality were inconsistent. Evidence from neurocognitive studies suggested structural and functional advantages, including larger total brain volume, improved white matter integrity, and enhanced cognitive performance, yet findings on memory and dementia outcomes remain inconclusive. Overall, this narrative review highlights global inequities in DHA intake and data availability, particularly in MICs, where national nutrition surveillance remains limited. These disparities may contribute to unequal protection against cardiovascular and neurocognitive decline. Strengthening dietary monitoring, improving access to DHA-rich or fortified foods, and promoting supplementation in vulnerable populations are key strategies to reduce inequities and support healthy aging worldwide.

## 1 Introduction

Fatty acids (FAs) play multiple physiological roles as energy sources, membrane phospholipid components, protein acylation donors, and precursors of lipid mediators. They are classified according to carbon chain length into short, medium, long, and very long chain FAs, and by double bond number into saturated, monounsaturated, and polyunsaturated fatty acids (PUFAs) (1). Among PUFAs, omega-3 fatty acids, particularly alpha-linolenic acid (ALA), eicosapentaenoic acid (EPA), and docosahexaenoic acid (DHA), are considered essential nutrients, since mammals lack the enzymes to synthesize them *de novo* and must obtain them from dietary sources (1, 2). Beyond their structural role, EPA and DHA act as precursors of specialized pro-resolving lipid mediators such as resolvins, protectins, and maresins, and as ligands of nuclear receptors like PPARα, thereby lowering circulating triglycerides and contributing to cardioprotective effects often linked to fish oil consumption (1, 2). DHA is of particular relevance due to its high molecular flexibility, which imparts unique properties to glycerophospholipids (GPLs) in membranes, influencing fluidity and dynamic processes such as fusion and fission (1). While the precise mechanisms of its incorporation remain unclear, DHA is especially abundant in the retina, brain, heart, testes, and skeletal muscle, where it fulfills key functions. In the central nervous system, DHA has been associated with neurodevelopment, cognitive function, and disease prevention (1, 2). Clinical and epidemiological evidence suggest that higher intake of long-chain omega-3, with emphasis on DHA, promotes cardiovascular and cognitive health (3). In animal models, DHA is preferentially incorporated into myocardial phospholipids compared with EPA, and in humans, DHA supplementation, but not EPA, has been shown to effectively lower heart rate, highlighting distinct biological roles (1).

At the international level, dietary recommendations for DHA and EPA vary considerably. Some national authorities provide guidance on long-chain omega-3 polyunsaturated fatty acids. For example, dietary reference frameworks developed in Japan emphasize adequate intake of long-chain omega-3 fatty acids for cardiometabolic and cognitive health, with practical consumption targets often cited in the literature around 2 g/day of total omega-3 s, although formal reference values for DHA specifically are not explicitly defined (4). Similarly, the World Health Organization recommends that 1–2 % of total daily energy be derived from polyunsaturated fatty acids (5). In the United States, Australia, and China, guidelines range from 250 to 2000 mg per day of combined EPA and DHA without establishing a minimum DHA requirement. France stands out as an exception, where the French Food Safety Agency recommends 250 mg per day of DHA specifically. These differences reflect disparities in evidence availability, public health priorities, and nutritional policy (6, 7). According to the National Institutes of Health (NIH), infants aged 0 to 12 months should consume 100 mg per day of DHA to support growth and neurodevelopment, while adults are advised to consume at least 250 mg per day of combined EPA and DHA. For pregnant and lactating women, a minimum of 200 mg per day of DHA is recommended to optimize maternal and infant outcomes (6, 7).

Although humans can endogenously convert ALA, present in plant oils, into DHA through elongate and desaturase activity, this process is highly inefficient, with conversion rates ranging from 0.01 to 10 percent. In healthy young women, only about 9% of dietary ALA is converted to DHA, while in young men, this conversion is practically nonexistent, and diets high in pro-inflammatory omega-6 fatty acids (common in Western dietary patterns) can further decrease conversion efficiency by up to 40% (8, 9). Reported ALA intakes vary widely across countries and income levels: in high-income countries (HICs) such as the United States, ALA represents up to 1.06% of total energy, whereas in Bulgaria it accounts for only 0.06%. In low-income countries (LICs), lower middle-income countries (LMICs) and upper middle-income countries (UMICs) such as Mexico, reported intakes are slightly higher (0.14%). These differences likely reflect disparities in access to ALA-rich foods, cultural variations in dietary patterns, and the economic availability of plant-based products or supplements containing omega-3 (9, 10).

Therefore, exogenous intake remains the primary determinant of DHA status. Coldwater fatty fish such as salmon, mackerel, herring, and trout provide approximately 0.68 to 1.43 g of DHA per 100 g, while canned sardines may supply up to 1.2 g per 100 g. By contrast, terrestrial meats like pork and chicken contain negligible levels (0.002 to 0.02 g per 100 g). Despite the availability of these sources, global intake remains inadequate. It is estimated that two-thirds of the adult population consume less than 250 mg per day of long-chain omega-3 fatty acids, substantially below recommended levels (8). Several factors influence these patterns, including geographical location (coastal vs. inland), dietary habits, socioeconomic context, and concerns about marine contamination with heavy metals, persistent organic pollutants, and methylmercury. Dietary patterns also play a significant role in determining tissue DHA levels; for instance, lower DHA concentrations have been observed in the adipose tissue of vegans and ovo-lacto-vegetarians compared to omnivorous individuals (9). Stratification of intake by gross national income (GNI) further highlights global disparities. Estimates indicate that in LIC and LMICs, mean DHA intake often falls below 50 mg per day, whereas in UMICs it averages around 43 mg per day. In contrast, some HICs, particularly those with strong fish consumption traditions such as Norway and Japan, frequently report intakes above 250 mg per day (11, 12). These differences also translate into variable blood levels of DHA and EPA across populations.

While there is a significant body of evidence for numerous adult health outcomes associated with DHA consumption, there remains variability among study findings. A number of both epidemiologic and clinical studies report that greater intake of DHA was associated with lower risk for cognitive decline (13), heart disease and inflammation (14, 15); however, other studies failed to demonstrate a statistically significant benefit, or produced varying results based upon sex, age, dosage, or duration of intervention (16–18). As a result, meta-analysis and randomized control trials typically produce different conclusions regarding DHA’s benefit due to differing methodologies, baseline nutritional status of participants and/or source (dietary vs. supplement) of DHA (19–21). Given the physiological changes that occur during aging, the potential neuroprotective and cardiometabolic effects of DHA may be particularly relevant in older adults; however, the replicability of these potential benefits has not been consistent across populations (21–23).

While evidence in children and maternal populations from LICs, LMICs and UMCs documents limited DHA intake (12, 24–28), there is a lack of comprehensive studies in adults that compare intake and functional outcomes such as cognition and cardiovascular health between low- and high-income regions. To our knowledge, this review represents the first narrative synthesis specifically documenting the associations of DHA intake with adult and older adult health, while explicitly contrasting countries by gross national income classification. The objective of this review is to synthesize the available evidence on DHA intake-related exposures, biomarkers, and health outcomes in adults and older adults across countries of different income levels, with the aim of identifying disparities, characterizing evidence gaps, and discussing how these differences may inform the development of targeted and context-specific public health strategies for vulnerable populations worldwide.

## 2 Materials and methods

We developed a comprehensive search strategy in PubMed using MeSH terms such as “DHA,” “Health,” “Dietary Patterns,” and “Developing Countries,” in order to capture studies conducted across different economic settings, particularly LIC and LMIC. We also reviewed reference lists of relevant articles, including narrative and systematic reviews, to identify additional studies addressing DHA intake and health-related outcomes. We used Covidence software to manage the review process, including duplicate removal, title and abstract screening, full-text selection, and data extraction (29). Three reviewers (BA-G, BV-V, and XT-L) independently screened the studies, and a third author (AG-R) resolved discrepancies when necessary.

We included studies that examined DHA as exposure, either through dietary intake or supplementation, or through biochemical assessment in biological matrices such as red blood cells, plasma, adipose tissue, semen, etc. The outcomes of interest included cardiovascular health, cognitive function, mental health, liver conditions, cancer risk, immune response, vegetarian dietary patterns, among others health related outcomes. For organizational purposes, DHA-related outcomes identified across studies were categorized by health domain and life stage to improve clarity and readability of the review, as presented in Supplementary Table 1. Eligible studies included original research articles, such as randomized controlled trials (RCT), cohort studies, case–control studies and cross-sectional studies, as well as narrative reviews, systematic reviews and meta-analyses, published between 2014 and 2026 in English or Spanish. The literature search was initially conducted up to mid-2025 and subsequently updated to include all relevant publications available through the end of 2025 and into early 2026. Countries were grouped by income level according to the World Bank’s fiscal year 2026 classification, based on 2024 GNI per capita using the Atlas method: low-income (≤$1,135), lower-middle-income ($1,136–$4,495), upper-middle-income ($4,496–$13,935), and high-income (≥$13,935). Extracted information was synthesized narratively and interpreted in relation to health outcome domains and national income level.

## 3 Results

A total of 2,621 records were identified. After screening and full-text evaluation, 52 studies met the eligibility criteria and were included in the narrative synthesis (Supplementary Figure 1). The search strategy initially covered publications up to mid-2025 and was later updated to capture studies published during late 2025 and early 2026, resulting in the inclusion of ten additional studies. Of the 52 included studies, 43 were conducted in HIC, eight in UMIC, and one provided global estimates with cross-country comparisons of DHA intake and availability (Tables 1–3). For evidence syntheses not restricted to a single country, GNI classification reflects the predominant income level of the primary studies included. We included the following studies: one systematic review (30), one prospective meta-analysis (31) and one individual-participant meta-analysis (32), 16 narrative reviews (33–48), three clinical trials (49–51), 13 cohorts (52–64), two case-cohort study (65, 66), two case–controls (67, 68), 12 cross-sectional studies (69–80) and one ecological study design (11).

**Table 1:** Synthesis of evidence on DHA intake, biomarkers, and cardiovascular health outcomes in adults, stratified by national income level.

Author, year, country	GNI	Study design	Population	Health outcome domain	Reported intake and/or biomarkers	Main health outcomes related to DHA
Ament (59), 2024, United States	High income	Prospective cohort (case-cohort analysis; REGARDS study)	Adults ≥45 years (REGARDS cohort); analytic sample: 1,075 incident ischemic stroke cases and 968 subcohort participants	Cardiovascular disease	Biomarkers: Plasma DHA-related lipid factor derived from lipidomic profiling (LC–MS/MS). Dietary intake: Fish intake (g/day) and dietary DHA estimated by FFQ.	Higher plasma DHA lipid factor was associated with a lower risk of incident ischemic stroke (HR = 0.88; 95% CI: 0.83–0.94), with stronger associations observed in women.
Amigó (66), 2020, United States	High income	Cross sectional	Adults (*n* = 26,034; men and women aged 30–70 years)	Cardiovascular disease	Dietary intake: Fish and seafood consumption assessed by FFQ, including salmon, canned tuna, dark- and light-fleshed fish, shrimp, lobster, and scallops. Biomarkers: Plasma DHA measured as part of total fatty acid composition in plasma phospholipids (μmol/L).	Higher DHA intake was positively associated with large LDL particle concentration (*β* = 0.023; 95% CI: 0.016–0.030; *p* < 0.001). Higher DHA intake was positively associated with large HDL particle concentration (*β* = 0.012; 95% CI: 0.009–0.016; *p* < 0.001). No significant association was observed between DHA intake and small LDL or small HDL particles (*p* > 0.05).
Bazinet (36), 2014, Multiple countries	High income	Narrative Review	General population	Cardiovascular disease	Biomarkers: DHA discussed as a major structural component of brain phospholipids; circulating DHA supply from blood to brain; DHA transport mechanisms (e.g., lysophospholipid-DHA via Mfsd2a). Dietary intake: DHA intake discussed qualitatively; no quantitative dietary intake data reported. Fish and seafood sources.	Higher RBC DHA was inversely associated with fasting triglycerides (*β* = −0.18; *p* < 0.001). RBC DHA was positively associated with LDL particle size (*β* = +0.21; *p* < 0.001). No significant association was observed between RBC DHA and total LDL-cholesterol (*p* > 0.05).
Chen (61), 2023, United States	High income	Prospective cohort study (NHANES linked to National Death Index)	Hypertensive adults ≥18 years; *n* = 26,914; median follow-up 8.6 years; 6,333 all-cause deaths and 2,039 cardiovascular deaths	Cardiovascular disease	Dietary intake: Median dietary DHA intake: 0.019 g/day (IQR 0.002–0.061 g/day). Participants categorized into low vs. high DHA intake groups using the median cutoff. Biomarkers: No circulating or tissue DHA biomarkers were measured.	Higher dietary DHA intake was associated with lower all-cause mortality (high vs. low intake: adjusted HR 0.95; 95% CI: 0.90–0.99; *p* < 0.05). Cardiovascular mortality: No statistically significant association between dietary DHA intake and cardiovascular mortality after multivariable adjustment (adjusted HR 0.98; 95% CI: 0.89–1.07). Non-linear analysis: Lowest all-cause mortality risk observed at dietary DHA intake of approximately 2.36 g/day; no significant protective range identified for cardiovascular mortality.
De Groot (30), 2019, Multiple countries	High income	Systematic review	Adults (systematic review of adult studies); multiple cohorts/clinical samples across included publications	Cardiovascular disease	Biomarkers: DHA assessed in plasma and erythrocyte fatty acids (wt% of total fatty acids), frequently within the omega-3 index framework (erythrocyte EPA + DHA). Dietary intake: Not the primary exposure; no pooled DHA intake (mg/day) reported. Genetics/Conversion: ELOVL2 polymorphisms associated with ~6% lower DHA; ALA increases EPA but not DHA. DHA levels influenced by intake of preformed DHA; ALA intake did not increase DHA levels. Supplement form (triglyceride vs. ethyl ester) affected DHA incorporation into erythrocytes.	Higher DHA levels reported in women (plasma DHA + 0.12% vs. men) and with increasing age (plasma EPA + DHA ~ +38% from 20–79 y; erythrocytes ~ + 19% from 20–75 y). Lower DHA levels reported with smoking (−6 to −17%) and higher BMI at low omega-3 index (≤5.6%); no BMI association when >7%. Wine consumption positively related to n-3 LCPUFA levels (plateau ~2–3 glasses/day).
Djuricić (44), 2025, Serbia	Upper middle income	Narrative review	Adults and older adults. Primarily human studies, with supportive evidence from animal and *in vitro* models	Cardiovascular disease	Dietary intake: DHA intake is discussed qualitatively through fish consumption and DHA-containing supplements. The review does not report original quantitative intake data. Biomarkers: DHA status is referenced via plasma and erythrocyte membrane DHA levels from cited studies as indicators of long-term exposure. No original biomarker measurements are generated.	DHA is described as having cardioprotective and anti-atherogenic effects. Higher DHA exposure is associated, in cited studies, with improved endothelial function, reduced arterial stiffness, and lower blood pressure. DHA contributes to anti-inflammatory effects relevant to cardiovascular disease through modulation of cytokines and lipid mediators. Effects are described as dose-dependent and heterogeneous, varying across study designs and populations.
Domenichiello (39), 2015, Multiple countries	High income	Narrative review	Humans and animal models (review synthesizing evidence on DHA metabolism, tissue accretion, and turnover across the life course; adult data included)	Cardiovascular disease	Dietary intake: DHA intake from diet and supplements is discussed qualitatively; no pooled or population-representative intake values are reported. Biomarkers: DHA is quantified in plasma phospholipids, erythrocytes, brain, heart, and other tissues across cited studies; tissue DHA levels depend mainly on preformed DHA intake rather than endogenous synthesis. Metabolism: Human conversion of ALA to DHA is very low (<1%); DHA turnover is slow in brain and heart and faster in circulating compartments	DHA is described as a critical structural component of cell membranes in metabolically active tissues, particularly the brain and heart, where it accumulates preferentially. The review reports that dietary DHA is required to maintain tissue DHA levels, as endogenous synthesis from ALA is insufficient to sustain physiological demands. Low DHA intake is described as leading to reduced DHA accretion in target tissues, whereas increased dietary DHA results in dose-dependent increases in tissue DHA, particularly in plasma and erythrocytes, with slower but measurable increases in cardiac and neural tissues. DHA is described as influencing membrane fluidity, lipid signaling, and inflammatory resolution pathways, providing a mechanistic basis for observed cardiovascular and systemic health effects reported in the broader literature.
Jakobsen (56), 2017, Denmark	High income	Cohort study	Adults aged 50–64 years at baseline; participants of the Danish Diet, Cancer, and Health cohort Analytical sample: 29,152 participants Subcohort with adipose tissue biopsies: 1,660 participants	Cardiometabolic health	Median dietary DHA intake: 0.39 g/day (80% central range: 0.17–0.77 g/day). Adipose tissue DHA content: 0.27% (0.15–0.45%) of total fatty acids.	Dietary DHA intake showed no consistent or statistically significant association with 5-year change in body weight or waist circumference when analyzed in quintiles. In linear models, a 250 mg/day higher DHA intake was associated with a small increase in body weight (*β* = 58.8 g; 95% CI: 0.8–116.7 g), with no association with waist circumference. Adipose tissue DHA content was not associated with 5-year change in body weight or waist circumference across quintiles (P for trend > 0.05). Overall, DHA intake and adipose tissue DHA were not appreciably associated with long-term changes in body weight or central adiposity.
Jehi (49), 2022, United States	High income	Randomized controlled trial	Older adults aged 63–79 years (mean age 69 ± 3 years), cognitively healthy; sub-sample from the Loma Linda center of the Walnuts and Healthy Aging (WAHA) study Analytical sample: 192 participants	Cardiovascular disease	Dietary intake: Baseline dietary intake of long-chain omega-3 fatty acids (EPA + DHA) was low (≈ 160 mg/day); dietary DHA was not reported separately and did not change during the 1-year intervention. Biomarkers: Erythrocyte DHA (% of total fatty acids) was measured independently. Over 1 year, DHA decreased in the walnut group (–0.125%) and increased in the control group (+0.174%), with a significant between-group difference (*p* = 0.004).	Walnut consumption for 1 year resulted in a small but statistically significant decrease in erythrocyte DHA, whereas DHA increased slightly in the control group. No improvement in n-3 index was observed, indicating that increased dietary ALA did not translate into higher DHA status. When both groups were combined, higher n-3 index (driven partly by DHA) was inversely associated with cardiometabolic risk factors: Total cholesterol: *β* = –5.59 mg/dL per 1% increase in n-3 index (*p* = 0.010) Triglycerides: *β* = –10 mg/dL (*p* = 0.010); Fasting plasma glucose: *β* = –0.27 (*p* = 0.013); No significant associations were observed with LDL-cholesterol, HDL-cholesterol, blood pressure, or body weight.
Okai-Mensah (47), 2025, United Kingdom	High income	Narrative review	Adults and older adults Evidence synthesized from human observational studies, randomized controlled trials, and mechanistic studies	Cardiometabolic health	DHA intake discussed qualitatively from dietary sources and supplements; no pooled or population-level intake values reported. DHA status referenced via plasma, serum, erythrocyte membrane, and tissue phospholipid levels; endogenous ALA→DHA conversion is described as very low, with tissue levels depending mainly on preformed DHA intake.	DHA is discussed in relation to lipid metabolism and inflammation within cardiometabolic health. Higher DHA intake or status is associated, in cited studies, with lower triglyceride concentrations, particularly at higher doses. Evidence for effects on glucose metabolism and insulin sensitivity is inconsistent. Overall, findings are described as dose-dependent and heterogeneous, without definitive causal conclusions.
Shi (63), 2025, Multiple countries (Europe, including the United Kingdom)	High income	Prospective cohort study	Adults (172,891) without prior vascular disease at baseline from: EPIC-CVD, UK Biobank, and INTERVAL.	Cardiovascular disease	Dietary intake: Not the primary exposure; diet is referenced only via correlation analyses (fish intake correlated with DHA). Biomarkers: Circulating DHA expressed as percentage of total fatty acids. DHA was among the fatty acids measured in all three cohorts (plasma phospholipids in EPIC-CVD; total plasma or serum in UK Biobank and INTERVAL).	Coronary heart disease (CHD): Higher circulating DHA was inversely associated with CHD risk. In the fully adjusted model (including lipid markers), per 1-SD increase in DHA: HR 0.88 (95% CI 0.84–0.93). Stroke: Circulating DHA showed no association with stroke risk across all models. In the fully adjusted model: HR 1.00 (95% CI 0.90–1.10) Circulating DHA was inversely associated with incident coronary heart disease but showed no association with stroke risk.
Solomons (80), 2015, Guatemala	Upper middle income	Cross-sectional study	Adult women: *n* = 158, age 18–48 years (mean ≈ 29–30 years) Schoolchildren: *n* = 135, age 6–11 years (mean ≈ 8 years) Rural communities in the Pacific coastal plain of Guatemala (Retalhuleu)	Cardiometabolic health/Nutritional status	Dietary intake: Not directly assessed in this study. Biomarkers: Erythrocyte DHA (% of total fatty acids) measured by gas–liquid chromatography. Mean erythrocyte DHA: All participants: 3.27 ± 0.66% Adult women: 3.09 ± 0.71% Schoolchildren: 3.49 ± 0.50% Omega-3 Index (EPA + DHA) median for the total sample: 3.58%, below the proposed cardioprotective threshold.	Findings indicate low erythrocyte DHA status in both Guatemalan women and schoolchildren, reflecting low long-chain n-3 PUFA availability. Compared with reference data from high-income countries (e.g., U.S. and European cohorts), erythrocyte DHA levels in this population were substantially lower, suggesting potential increased cardiometabolic risk based on biomarker thresholds. Differences in DHA status between women and children were modest; children showed slightly higher erythrocyte DHA than women.
Valera (71), 2014, French Polynesia	High income	Cross-sectional study	Adults ≥18 years from French Polynesia with high habitual fish intake.	Cardiovascular health	Biomarkers: DHA measured in erythrocyte membrane phospholipids (% of total fatty acids). Mean DHA: 6.41 ± 1.40%. Dietary intake not directly quantified (biomarker-based exposure).	Resting heart rate: Inversely associated with DHA (*β* = −2.57 bpm per 1% DHA; *p* = 0.005). Diastolic blood pressure: Inversely associated with DHA (*β* = −1.96 mmHg; *p* = 0.05). Heart rate variability: Higher DHA associated with increased parasympathetic indices (HF and rMSSD). No significant association observed with systolic blood pressure.
Valookaran (35), 2022, Multiple countries	High income	Narrative review	Adults (human trials summarized include adults with: overweight/hyperlipidemia, treated hypertension with type 2 diabetes; also summarizes preclinical models)	Cardiovascular Disease	No original intake or biomarker data are generated in this review. DHA is discussed as a long-chain n-3 PUFA found in fish oil and other food sources (review statement). Human evidence summarized is primarily from supplementation trials with DHA (g/day) and blood pressure outcomes.	Human intervention trials summarized: 3 Dose and duration: DHA 2–4 g/day for 5–6 weeks. Blood pressure effects: 2 of 3 trials reported significant reductions in systolic and diastolic blood pressure with DHA supplementation in overweight or mildly hyperlipidemic adults. In these trials, EPA showed no significant effect, while DHA alone was associated with blood pressure reductions. Null findings: In adults with treated hypertension and type 2 diabetes, DHA supplementation (4 g/day) did not significantly affect blood pressure. Evidence for a DHA-specific antihypertensive effect is limited and inconsistent, with effects observed only in selected adult populations
Vazquez (51), 2014, Spain	High income	Multicenter Randomized crossover clinical trial	Adults with metabolic syndrome (*n* = 273; aged 30–70 y; 13 Spanish centers; 257 completed study)	Cardiovascular disease	Fish intake: 7 servings of hake (100 g each) per week. Serum DHA: significant increase (treatment effect *p* < 0.001)	Lipid profile: Decrease in LDL cholesterol (*p* = 0.048); no significant changes in HDL cholesterol or triglycerides. Blood pressure: Reduction in diastolic BP (*p* = 0.014). Anthropometry: Reduction in waist circumference (*p* < 0.001). Inflammation: No change in C-reactive protein. Serum fatty acids: Increase in EPA, DHA, and n-3/n-6 ratio (*p* < 0.001). Regular consumption of 100 g/day white fish for 8 weeks improved lipid profile, blood pressure, and waist circumference, while increasing serum DHA and EPA concentrations in adults with metabolic syndrome.
Veno (58), 2019, Denmark	High income	Prospective cohort study (Diet, Cancer and Health Study)	Adults (*n* = 55,338; 50–65 y at baseline); median follow-up = 13.5 y	Cardiovascular disease	Marine n-3 PUFA, EPA, DHA intake (g/d) and adipose-tissue content (% wt) categorized in quartiles (Q1–Q4).	Total ischemic stroke (*n* = 1,879): DHA intake Q4 vs. Q1: HR = 1.06 (95% CI: 0.94–1.21); *P* trend = 0.513. Adipose-tissue DHA Q4 vs. Q1: HR = 1.00 (95% CI: 0.83–1.20); *P* trend = 0.580. Large artery atherosclerosis (*n* = 319): DHA intake Q4 vs. Q1: HR = 0.72 (95% CI: 0.53–0.99); *P* trend = 0.043. Cardioembolism (*n* = 102): DHA intake Q4 vs. Q1: HR = 2.12 (95% CI: 1.21–3.69); *P* trend = 0.002. Small-vessel occlusion (*n* = 844): no association (DHA Q4 vs. Q1 HR = 1.13 [0.93–1.38]). Adipose-tissue DHA: Cardioembolism Q4 vs. Q1 HR = 2.00 (95% CI: 1.04–3.84); *P* trend = 0.030. DHA was inversely associated with large artery atherosclerosis and positively associated with cardioembolic stroke.
Yang (31), 2016, Multiple countries	High income	Meta-analysis of prospective cohort studies	Adults (*n* = 56,204) from 8 cohorts conducted in the United States, Europe, and Asia; 20,497 incident cases of elevated blood pressure (follow-up: 3–20 years).	Cardiovascular Disease	Dietary intake: Comparison of highest vs. lowest intake categories: - Fish consumption: SRR = 0.96 (95% CI: 0.81–1.14). - Total LC n-3 PUFA intake: SRR = 0.73 (95% CI: 0.60–0.89). - DHA dietary intake: SRR = 0.70 (95% CI: 0.30–1.63). Biomarkers: Circulating LC n-3 PUFA: SRR = 0.67 (95% CI: 0.55–0.83); DHA biomarker: SRR = 0.64 (95% CI: 0.45–0.89).	Blood pressure: No association with dietary DHA intake. Circulating DHA inversely associated with elevated BP: SRR = 0.64 (95% CI: 0.45–0.89). Total LC n-3 PUFA (biomarker): SRR = 0.67 (95% CI: 0.55–0.83). *Summary*: Higher circulating DHA levels associated with lower risk of elevated BP; no consistent effect observed for dietary intake.
Zeinalabedini (68), 2024, Iran	Upper middle income	Population-based case–control study	Cases: Adults aged 40–80 years; *n* = 433 with clinically confirmed ischemic heart disease (IHD) (≤3 months since diagnosis) Controls: Adults aged 40–80 years; *n* = 453, free of IHD, recruited from the same hospital	Cardiovascular disease	Dietary intake: Assessed using a validated semi-quantitative 237-item FFQ; Nutrient intake calculated with Nutritionist IV software. DHA intake (mg/day): Cases: 113.6 ± 12.58 mg/day; Controls: 141.9 ± 19.57 mg/day; *p* = 0.01 Biomarkers: No circulating or tissue DHA biomarkers were measured.	Outcome: Presence of ischemic heart disease (IHD). Crude and adjusted logistic regression models consistently showed an inverse association between dietary DHA intake and IHD. Fully adjusted model (Model 5): OR = 0.98 (95% CI 0.97–0.99), *p* = 0.04 The association remained statistically significant after adjustment for: age, sex, total energy intake, total fat intake, BMI, smoking, alcohol use, and physical activity. No significant associations were observed between IHD and intake of SFA, MUFA, PUFA, trans fatty acids, ALA, or EPA. Authors conclude that higher dietary DHA intake may be associated with a modestly lower risk of IHD, noting that the effect size is small and clinical relevance should be interpreted with caution.

GNI, Gross National Income; DHA, Docosahexaenoic Acid; EPA, Eicosapentaenoic Acid; ARA, Arachidonic Acid; PUFA, Polyunsaturated Fatty Acid; LC n-3 PUFA, Long-Chain Omega-3 Polyunsaturated Fatty Acids; FFQ, Food Frequency Questionnaire; RBC, Red Blood Cell; HR, Hazard Ratio; OR, Odds Ratio; CI, Confidence Interval; LDL, Low-Density Lipoprotein; HDL, High-Density Lipoprotein; VLDL, Very Low-Density Lipoprotein; BP, Blood Pressure; T2D, Type 2 Diabetes; IL, Interleukin; TNF-α, Tumor Necrosis Factor-alpha; CRP, C-Reactive Protein; PAI-1, Plasminogen Activator Inhibitor-1; ALA, Alpha-Linolenic Acid; MUFA, Monounsaturated Fatty Acid; SFA, Saturated Fatty Acid; NAFLD, Non-Alcoholic Fatty Liver Disease; IHD, Ischemic Heart Disease; RCT, Randomized Controlled Trial; LC–MS/MS, Liquid Chromatography–Tandem Mass Spectrometry; GC–MS, Gas Chromatography–Mass Spectrometry; MRI-PDFF, Magnetic Resonance Imaging–Proton Density Fat Fraction; NHANES, National Health and Nutrition Examination Survey; UK Biobank, United Kingdom Biobank; REGARDS, Reasons for Geographic and Racial Differences in Stroke; PLCO, Prostate, Lung, Colorectal, and Ovarian Cancer Screening Trial.

GNI classification follows the World Bank Atlas method for the 2026 fiscal year, based on 2024 GNI per capita.

DHA exposure was assessed using dietary intake estimates, biochemical biomarkers (e.g., plasma, erythrocytes, adipose tissue fatty acid composition), or other biological samples, as reported in each study.

Reported outcomes reflect associations between DHA intake or status and health-related indicators across multiple domains; causality should not be inferred unless supported by randomized controlled trial designs.

Methods used to assess DHA exposure and health outcomes varied across studies; therefore, direct quantitative comparisons should be interpreted with caution.

For narrative reviews, systematic reviews, and meta-analyses not restricted to a single country, national income classification reflects the predominant income level of the countries from which the evidence originates and is used for organizational purposes.

Adult and older adult populations include heterogeneous age ranges and health conditions, as defined in the original studies.

**Table 2:** Synthesis of evidence on DHA intake, biomarkers, and cognition and mental health outcomes in adults, stratified by national income level.

Author, year, country	GNI	Study design	Population	Health outcome domain	Reported intake and/or biomarkers	Main health outcomes related to DHA
Cardoso (41), 2016, Multiple countries	High income	Narrative review	Adults and older adults, including healthy individuals and those with mild cognitive impairment (MCI) or Alzheimer’s disease (AD)	Cognition and mental health	Dietary intake: DHA intake reported across included studies ranged from 0.2 to 2.0 g/day, mainly from fish oil or algal DHA supplements; habitual dietary DHA intake in Western populations described as low (~100 mg/day). Biomarkers: Lower blood DHA levels associated with cognitive decline during ageing; higher plasma or serum phospholipid DHA associated with better cognitive performance.	Higher DHA intake or status is associated with better cognitive performance, including memory, executive function, and working memory, particularly in ageing adults. Reduced DHA levels are associated with cognitive decline during ageing. DHA supplementation (≈ 0.9–2.0 g/day) improved memory and learning outcomes in several RCTs in healthy older adults and individuals with MC. Evidence for benefit in established Alzheimer’s disease is inconsistent, with potential benefit limited to ApoE4-negative individuals.
Chu (57), 2022, Taiwan	High income	Prospective cohort study (2-year follow-up)	Patients with Alzheimer’s disease aged ≥65 years receiving acetylcholinesterase inhibitors; *n* = 129 (45 men, 84 women); mean age 76.5 ± 6.6 years	Cognition and mental health	Dietary intake (DHA): Mean DHA intake: 465.3 ± 430.1 mg/day in the decline group vs. 480.3 ± 698.7 mg/day in the stable group (*p* = 0.981); no difference between groups. Biomarkers: Serum DHA: Decline group: 1.8 ± 3.6 mg/mL Stable group: 5.2 ± 7.1 mg/mL (*p* = 0.023)	Lower baseline serum DHA concentrations were associated with a higher risk of cognitive decline over 2 years in patients with Alzheimer’s disease receiving acetylcholinesterase inhibitors (adjusted OR 1.131; 95% CI: 1.020–1.254; *p* = 0.020). Dietary DHA intake was not associated with cognitive decline, either as CDR worsening or as continuous MMSE change (*p* > 0.05). DHA showed an independent association with cognitive decline risk after adjustment, whereas EPA did not.
De Oliveira Otto (54), 2018, United States	High income	Prospective cohort study	Community-dwelling adults from the ARIC cohort, free of dementia at baseline.	Cognition and mental health	Biomarkers: Plasma phospholipid DHA (% of total fatty acids), analyzed in quintiles and per interquartile range (IQR). Dietary intake: Not used as primary exposure.	Cognitive decline (3MSE): No significant association (*β* for annual change −0.03; *p* = 0.28). Incident cognitive impairment: No significant association across DHA quintiles (Q5 vs. Q1 HR 0.80; p-trend = 0.21); Total dementia: No significant association across DHA quintiles (Q5 vs. Q1 HR 1.01; p-trend = 0.74); Alzheimer’s disease: No significant association across DHA quintiles (Q5 vs. Q1 HR 0.96; p-trend = 0.45). Circulating DHA was not associated with cognitive decline, incident cognitive impairment, total dementia, or Alzheimer’s disease in this cohort.
Gustafson (55), 2020, United States	High income	Observational study	Adults aged ≥65 years	Cognition and mental health	Biomarkers: Plasma DHA (% of total fatty acids) measured and analyzed independently from EPA.	Dietary DHA intake was evaluated independently and increased across tertiles from 0.06 g/day (T1) to 0.11 g/day (T2) and 0.24 g/day (T3). Incident Alzheimer’s disease cases declined with increasing DHA intake (145 cases in T1, 118 in T2, and 115 in T3). Compared with the lowest tertile, higher DHA intake was associated with a lower risk of incident Alzheimer’s disease, with risk ratios of 0.79 (95% CI: 0.62–1.01) for T2 and 0.76 (95% CI: 0.59–0.97) for T3, and a significant linear trend (*p* = 0.0245). Age-adjusted and multivariable-adjusted models showed consistent inverse associations, with a multivariable-adjusted hazard ratio of 0.73 (95% CI: 0.57–0.95) for the highest versus lowest DHA intake tertile (P for trend = 0.0184).
Hashemi (67), 2020, Iran	Upper middle income	Case–control study	Adults aged 18–60 years, including individuals with stress and anxiety disorders and healthy controls	Cognition and mental health	Dietary intake: Dietary intake was assessed using a 28-item FFQ. No dietary food group, including fish intake, showed a significant association with stress or anxiety after adjustment. Biomarkers: Erythrocyte fatty acid composition was measured, and DHA was analyzed independently. Erythrocyte DHA (% of total fatty acids): Stress/anxiety group: 0.832 ± 0.507. Control group: 1.148 ± 0.594; *p* = 0.008	Individuals with stress and anxiety exhibited significantly lower erythrocyte DHA levels compared with controls. Higher erythrocyte DHA was inversely associated with stress and anxiety status. In multivariable-adjusted logistic regression models, DHA was associated with lower odds of stress/anxiety (adjusted OR = 0.159; 95% CI: 0.032–0.775; *p* = 0.023). The association remained significant after adjustment for age, BMI, socioeconomic status, physical activity, and total energy intake.
Heath (33), 2021, Multiple countries	High income	Narrative review	Human and experimental studies	Cognition and mental health	This review does not report original dietary intake data or biomarkers. Summarizes evidence from epidemiological, clinical, and experimental studies evaluating dietary DHA, plasma DHA, erythrocyte DHA, and brain DHA. Highlights heterogeneity in DHA biomarkers, including plasma, erythrocytes, adipose tissue, and brain tissue, across studies.	Epidemiological studies summarized in the review consistently report inverse associations between higher DHA status or intake and Alzheimer’s disease risk, although randomized trials of DHA supplementation have shown inconsistent results. The review emphasizes that DHA metabolism, storage in adipose tissue, and transport to the brain may modify observed associations between DHA intake and cognitive outcomes. Variability in DHA form, duration of exposure, baseline DHA status, and brain uptake mechanisms is identified as a key factor explaining mixed findings in Alzheimer’s disease prevention and treatment.
Lewis (45), 2025, United Kingdom	High income	Narrative review	Adults and older adults. Evidence summarized from human observational studies, randomized controlled trials, and mechanistic studies	Cognition and Mental Health	Dietary intake: DHA intake is discussed qualitatively from dietary sources and supplementation; no pooled or population-representative intake values are reported. Biomarkers: DHA status is described using plasma phospholipids, erythrocytes, cerebrospinal fluid, and brain tissue DHA levels from cited studies. Endogenous conversion from ALA is reported as minimal (<1%), with tissue DHA levels depending mainly on preformed DHA intake.	DHA is described as a key structural and functional component of the brain. Higher DHA exposure is associated, in cited studies, with better cognitive performance and lower risk of cognitive decline and dementia. DHA-related effects are linked to modulation of neuroinflammation and synaptic function. Evidence from intervention trials is heterogeneous, with more consistent findings in individuals with low baseline DHA status or early cognitive impairment.
Loong (73), 2023, United States	High income	Cross-sectional study	Cognitively normal older adults aged 63–90 years Mean age: 76.3 ± 8.3 years Sample size: *n* = 40	Cognition and Mental Health	Dietary intake of DHA was not assessed or quantified. Biomarkers: Red blood cell DHA (% of total fatty acids) measured from dried blood spots. Mean DHA: 2.6 ± 1.1% Range: 1.14–5.74% DHA was analyzed independently from EPA and the omega-3 index.	Cognitive outcomes: DHA showed a positive, borderline association with delayed verbal memory: RAVLT-Delayed Recall: r = 0.33, *p* < 0.10 No statistically significant associations were observed between DHA and immediate memory, processing speed, executive function, or working memory. Brain structure outcomes: DHA was significantly positively correlated with total white matter volume: White matter volume: r = 0.33, *p* < 0.05 No statistically significant associations were observed between DHA and hippocampal volume, entorhinal cortex volume, or frontal pole thickness.
Macaron (42), 2021, Multiple countries	High income	Narrative review	Cognitively healthy adults aged ≥45 years, including middle-aged and older adults; non-demented, free of neurological or psychiatric disease.	Cognition and Mental Health	No original data. The review summarizes studies assessing dietary DHA intake and blood DHA biomarkers (plasma phospholipid DHA and erythrocyte DHA), either individually or as part of omega-3 measures, depending on the primary study	Across the studies summarized, higher DHA intake or higher blood DHA levels were most frequently associated with better cognitive performance and greater brain structural integrity in adults and older adults. Reported associations include larger hippocampal volume, greater total gray matter and total brain volume, and lower white matter lesion burden in individuals with higher DHA status. Longitudinal observational studies summarized suggest that higher erythrocyte or plasma DHA may be associated with slower rates of brain atrophy in specific regions, although findings are region-specific. Overall, the evidence is heterogeneous, with several studies reporting null or mixed associations, particularly when DHA is analyzed separately from other omega-3 fatty acids, and no causal conclusions are drawn.
Minihane (46), 2025, United Kingdom	High income	Narrative review	Adult women, with emphasis on midlife and postmenopausal women; evidence synthesized from human observational studies and randomized trials.	Cognition and Mental Health	DHA intake discussed qualitatively from diet and supplements; no pooled intake values reported. DHA status referenced via plasma phospholipids, erythrocytes, CSF, and brain tissue; endogenous ALA→DHA conversion is very low (<1%), particularly after menopause.	DHA is a key structural and functional lipid in the brain, with particular relevance during the menopausal transition. Higher DHA intake or status is associated, in cited observational studies, with better cognitive performance, lower risk of cognitive decline, and fewer depressive symptoms, especially in women with low baseline DHA. Intervention trials show heterogeneous effects on cognition and mood, with more consistent benefits observed in early postmenopause and among women with low habitual DHA intake.
Mohajeri (40), 2015, Multiple countries	High income	Narrative review	Adults and older adults; cognitively normal adults and individuals with age-related cognitive decline (review of human observational and intervention studies)	Cognition and Mental Health	No original dietary intake data or biomarker measurements are reported. The review synthesizes evidence from studies assessing dietary DHA intake, plasma DHA, erythrocyte DHA, and brain DHA content, depending on study design. DHA is discussed independently from EPA in several of the summarized studies, particularly those focused on brain structure and function.	The review reports that higher DHA intake or higher blood DHA levels are frequently associated with better cognitive performance, particularly in domains related to memory and learning, in adults and older adults. Observational studies summarized indicate associations between higher DHA status and reduced risk of cognitive decline, as well as better maintenance of cognitive function with aging. Neuroimaging and mechanistic studies reviewed suggest that DHA is related to greater synaptic integrity, neuronal membrane fluidity, and brain structural preservation, which may underlie observed cognitive benefits. The review also notes that intervention trials show mixed results, with benefits more consistently observed in individuals with low baseline DHA status or early cognitive changes, and no causal conclusions are established.
Natacci (72), 2018, Brazil	Upper middle income	Cross-sectional analysis (baseline data from a population-based cohort: ELSA-Brasil)	Adults aged 35–74 years Final analytical sample: 12,268 participants	Cognition and Mental Health	Dietary intake assessed using a validated 114-item FFQ. DHA intake was quantified in g/day and analyzed in quintiles, adjusted for total energy intake. Median DHA intake: With anxiety disorders: 0.42 g/day (IQR 0.14–0.79) Without anxiety disorders: 0.46 g/day (IQR 0.20–0.88)	Higher dietary DHA intake was inversely associated with anxiety disorders. In fully adjusted logistic regression models (adjusted for sociodemographic factors, cardiovascular risk factors, total energy intake, diet quality, and depression), participants in the highest quintile of DHA intake had lower odds of anxiety disorders compared with the lowest quintile: OR = 0.82 (95% CI: 0.69–0.98). However, after adjustment for multiple comparisons, the association between DHA intake and anxiety disorders lost statistical significance
Nozaki (52), 2021, Japan	High income	Prospective population-based cohort study (JPHC Saku Mental Health Study)	Adults aged 45–64 years at baseline (midlife); Final analytical sample: 1,127 participants (468 men, 659 women). Cognitive assessment conducted 14–15 years later (mean age at follow-up ≈ 73 years).	Cognition and Mental Health	Dietary intake: Assessed using a validated food frequency questionnaire (FFQ) in 1995 and 2000; average intake calculated. DHA intake analyzed in quartiles, energy-adjusted (residual method). Median DHA intake across quartiles (mg/day): 351.4, 524.9, 684.9, 956.2.	Higher dietary DHA intake in midlife was significantly associated with a lower risk of dementia in later life. In fully adjusted logistic regression models, compared with the lowest DHA quartile, the odds of dementia were reduced in: Second quartile: OR 0.39 (95% CI: 0.18–0.84) Third quartile: OR 0.30 (95% CI: 0.13–0.70) Highest quartile: OR 0.28 (95% CI: 0.12–0.66) (P for trend < 0.01). DHA intake was not significantly associated with mild cognitive impairment (MCI). Results were consistent after adjustment for age, sex, education, smoking, alcohol intake, physical activity, cardiovascular history, diabetes, and depression.
Qiu (79), 2025, United States	High income	Cross-sectional study	Adults ≥18 years from the general U.S. population (NHANES).	Cognition and Mental Health	Dietary intake: DHA intake estimated from two 24-h dietary recalls and expressed as daily intake. DHA analyzed in quartiles. Biomarkers: None reported.	Outcome: Depression (PHQ-9 ≥ 10). Highest vs. lowest DHA intake quartile: OR = 0.91 (95% CI: 0.86–0.96) after propensity score matching and full adjustment. Dose–response analysis: Significant inverse non-linear association between DHA intake and depression risk (P for non-linearity <0.05), with risk reduction plateauing at higher intakes. Subgroup analyses: No significant effect modification by sex, age, smoking status, alcohol consumption, or comorbidities (P for interaction >0.05).
Reyes-Barrera (76), 2022, Mexico	Upper middle income	Cross-sectional study	Apparently healthy adults (*n* = 36; 69% women; mean age 56 ± 8 y; BMI 26 ± 4 kg/m^2^)	Cognition and Mental Health	DHA intake: 0.09 (0.06–0.13) g/day [median (IQR)]. Additional variables: n-3 PUFA 1.2 (0.8–1.7) g/day; EPA 0.05 (0.02–0.06) g/day.	Glucose metabolism: log DHA inversely associated with log insulin (*β* = –0.318 ± 0.133; *p* = 0.02). HOMA-IR (*β* = –0.370 ± 0.140; *p* = 0.01); C-peptide (*β* = –4.4 ± 1.4; *p* = 0.006). Adipose tissue function: log DHA inversely associated with log resistin (*β* = –0.740 ± 0.192; *p* = 0.001). Marginal association with PAI-1 (*β* = –1.5 ± 0.8; *p* = 0.06). Higher DHA intake was associated with improved insulin sensitivity and lower resistin levels in Mexican adults.
Román (37), 2019, Spain	High income	Narrative review	Adults and older adults; human studies including cognitively healthy individuals and populations with cognitive impairment (review of observational and intervention studies)	Cognition and Mental Health	The review does not report original dietary intake data or biomarker measurements. It summarizes evidence from human studies evaluating dietary DHA intake, plasma DHA, erythrocyte DHA, and brain DHA levels, depending on the study cited. DHA is discussed independently from EPA in sections addressing neuronal membrane composition, synaptic function, and neuroinflammation, although some summarized studies assess DHA as part of combined omega-3 measures	The review reports that DHA is a major structural component of neuronal membranes, with high concentrations in the cerebral cortex and synaptic membranes, and is involved in synaptic plasticity and neurotransmission. Human observational studies summarized indicate that higher DHA intake or higher DHA status is associated with better cognitive performance and lower risk of cognitive decline in adults and older adults. The authors notes that lower DHA levels have been observed in individuals with cognitive impairment and Alzheimer’s disease compared with cognitively healthy controls. Evidence from intervention studies summarized is described as inconsistent, with cognitive benefits more frequently reported in individuals with low baseline DHA status or at early stages of cognitive decline, and no definitive causal conclusions are draw
Sala-Vila (60), 2023, United Kingdom	High income	Prospective population-based cohort study	Adults aged 40–69 years at baseline (UK Biobank) Total analytical sample: ≈267,000 participants Follow-up: median 8.8 years	Cognition and Mental Health	Dietary intake: Not used for DHA exposure. Biomarkers: Plasma DHA (% of total fatty acids), analyzed in quintiles: Q1: <1.46 (median 1.24) Q2: 1.46–1.78 (median 1.63) Q3: 1.78–2.07 (median 1.92) Q4: 2.07–2.47 (median 2.25) Q5: >2.47 (median 2.84)	Incident Alzheimer’s disease (AD): Overall population: No significant difference between Q5 and Q1 (HR 0.93 [0.81–1.06]); Age 50–59 years: Q5 showed a significantly lower risk compared with Q1 (HR 0.59 [0.37–0.94]); Age ≥60 years: No significant difference between Q5 and Q1 (HR 0.99 [0.85–1.15]). Incident all-cause dementia: Overall population: Q5 had a lower risk compared with Q1 (HR 0.87 [0.79–0.95]); Men: Q5 vs. Q1 HR 0.87 [0.77–0.99]; Women: Q5 vs. Q1 HR 0.84 [0.73–0.96]; Age 50–59 years: No significant difference for Q5 vs. Q1 (HR 0.94 [0.68–1.30]); Age ≥60 years: Q5 showed a modestly lower risk compared with Q1 (HR 0.89 [0.81–0.98]).
Wendołowicz (48), 2018, Poland	High income	Narrative review	Adults (human evidence discussed) The review also summarizes findings from animal and in vitro studies	Cognition and Mental Health	Dietary intake: DHA intake is discussed qualitatively, with fish and seafood identified as the primary dietary sources. No quantitative intake data (mg/day) are reported. Biomarkers: DHA status is referenced through serum, erythrocyte membrane, and brain tissue DHA levels from cited studies. Lower DHA concentrations in serum and erythrocytes are reported in individuals with neuropsychiatric disorders.	DHA is described as a key structural component of neuronal membranes, accounting for a substantial proportion of fatty acids in brain tissue. Low DHA status is consistently associated, across cited studies, with neuropsychiatric and cognitive disorders, including depression and schizophrenia; DHA-derived mediators (e.g., neuroprotectin D1) are described as contributing to neuroprotection, neuronal survival, and resistance to oxidative and inflammatory damage; DHA is reported to modulate neurotransmission, particularly serotonergic pathways, with reduced DHA linked to lower cerebrospinal fluid 5-HIAA concentrations. The review summarizes observational evidence suggesting higher DHA intake or fish consumption is associated with lower risk of cognitive decline and Alzheimer’s disease, while emphasizing that evidence is largely observational and mechanistic, not causal

GNI, Gross National Income; DHA, Docosahexaenoic Acid; EPA, Eicosapentaenoic Acid; ARA, Arachidonic Acid; PUFA, Polyunsaturated Fatty Acid; LC n-3 PUFA, Long-Chain Omega-3 Polyunsaturated Fatty Acids; FFQ, Food Frequency Questionnaire; RBC, Red Blood Cell; HR, Hazard Ratio; OR, Odds Ratio; CI, Confidence Interval; LDL, Low-Density Lipoprotein; HDL, High-Density Lipoprotein; VLDL, Very Low-Density Lipoprotein; BP, Blood Pressure; T2D, Type 2 Diabetes; IL, Interleukin; TNF-α, Tumor Necrosis Factor-alpha; CRP, C-Reactive Protein; PAI-1, Plasminogen Activator Inhibitor-1; ALA, Alpha-Linolenic Acid; MUFA, Monounsaturated Fatty Acid; SFA, Saturated Fatty Acid; NAFLD, Non-Alcoholic Fatty Liver Disease; IHD, Ischemic Heart Disease; RCT, Randomized Controlled Trial; LC–MS/MS, Liquid Chromatography–Tandem Mass Spectrometry; GC–MS, Gas Chromatography–Mass Spectrometry; MRI-PDFF, Magnetic Resonance Imaging–Proton Density Fat Fraction; NHANES, National Health and Nutrition Examination Survey; UK Biobank, United Kingdom Biobank; REGARDS, Reasons for Geographic and Racial Differences in Stroke; PLCO, Prostate, Lung, Colorectal, and Ovarian Cancer Screening Trial.

GNI classification follows the World Bank Atlas method for the 2026 fiscal year, based on 2024 GNI per capita.

DHA exposure was assessed using dietary intake estimates, biochemical biomarkers (e.g., plasma, erythrocytes, adipose tissue fatty acid composition), or other biological samples, as reported in each study.

Reported outcomes reflect associations between DHA intake or status and health-related indicators across multiple domains; causality should not be inferred unless supported by randomized controlled trial designs.

Methods used to assess DHA exposure and health outcomes varied across studies; therefore, direct quantitative comparisons should be interpreted with caution.

For narrative reviews, systematic reviews, and meta-analyses not restricted to a single country, national income classification reflects the predominant income level of the countries from which the evidence originates and is used for organizational purposes.

Adult and older adult populations include heterogeneous age ranges and health conditions, as defined in the original studies.

**Table 3:** Synthesis of evidence on DHA intake, biomarkers, and other health outcomes in adults, including cancer risk, dietary patterns, immune function, and liver disease, stratified by national income level.

Author, year, country	GNI	Study design	Population	Health outcome domain	Reported intake and/or biomarkers	Main health outcomes related to DHA
Bassett (65), 2016, Australia	High income	Prospective case–cohort study	Adults (women; *n* = 2,021 subcohort; 470 incident breast cancer cases)	Cancer risk	Dietary intake: DHA (g/day), EPA, DPA estimated by validated FFQ (energy-adjusted). Biomarker: DHA (% of total fatty acids) measured in plasma phospholipids.	DHA is required for normal brain development and maintenance of neuronal membrane structure. DHA modulates neurotransmission and synaptic function. DHA regulates neuroinflammatory processes in the central nervous system. Altered DHA-related pathways are associated with impaired cognitive function and mood disorders. Dysregulation of DHA metabolism is linked to neurodegenerative conditions, including Alzheimer’s disease.
Brasky (53), 2014, United States	High income	Prospective cohort study (VITamins and Lifestyle [VITAL] cohort)	Women aged 50–76 years; 22,494 participants; 263 incident invasive endometrial cancer cases identified after a median follow-up of 9 years.	Cancer risk	Dietary intake: EPA, DHA y EPA + DHA (mg/day) from diet assessed by FFQ and categorized into energy-adjusted quintiles; highest dietary EPA + DHA intake ≈ 165–256 mg/day. Supplements: EPA and DHA intake from diet plus supplements estimated; dose assumed from commercial fish-oil formulations.	Higher dietary DHA intake was associated with increased endometrial cancer risk (highest vs. lowest quintile: HR 1.66; 95% CI: 1.09–2.55; P-trend = 0.036). The association was modified by BMI: Normal-weight women (BMI < 25 kg/m^2^): higher dietary DHA intake associated with lower risk (highest vs. lowest quintile: HR 0.35; 95% CI: 0.14–0.85). Overweight/obese women (BMI ≥ 25 kg/m^2^): higher dietary DHA intake associated with higher risk (highest vs. lowest quintile: HR 2.60; 95% CI: 1.53–4.40).
Brasky et al. (32), 2023, Multiple countries	High income	Individual-participant data meta-analysis of prospective cohort studies	Women; 12 prospective cohort studies; 7,212 endometrial cancer cases and 26,031 controls	Cancer risk	Dietary intake: Docosahexaenoic acid (DHA) intake estimated from FFQs; energy-adjusted, study-specific quartiles (mg/day).	Higher dietary DHA intake was associated with a higher risk of endometrial cancer (highest vs. lowest quartile: OR 1.09; 95% CI: 1.01–1.19; P-trend = 0.04). The positive association was stronger among women with BMI ≥ 25 kg/m^2^; no protective association was observed.
Burdge (38), 2017, Multiple countries	High income	Narrative review	Adult women (vegetarian and vegan), including non-pregnant and pregnant women; evidence synthesized from multiple observational and metabolic studies in adults.	Vegetarian dietary patterns	Dietary intake: Vegetarian and vegan women consistently report very low or negligible DHA intake. Reported DHA intake in vegetarian women is <1 mg/day, and undetectable in vegan women, compared with omnivores (≈40–150 mg/day). Biomarkers: Vegetarian and vegan women show lower DHA concentrations in plasma, serum, erythrocyte phospholipids, breast milk, placental tissue, and umbilical cord blood compared with omnivores (≈15–69% lower, depending on matrix).	Vegetarian and vegan diets are consistently associated with lower DHA status in adult women compared with omnivorous diets. Endogenous conversion of *α*-linolenic acid to DHA is insufficient to compensate for the absence of pre-formed dietary DHA. Low maternal DHA status is associated with reduced DHA transfer to the fetus and infant, evidenced by lower DHA levels in cord blood, breast milk, and infant erythrocytes
Calder, 2026 (44), United Kingdom	High income	Narrative review (state-of-the-art review)	Adults and older adults The review synthesizes evidence from: Randomized controlled trials; Observational studies; Mechanistic and translational studies	Immune system function	Dietary intake: DHA intake is discussed through fish/fish oil consumption and supplementation studies; no original quantitative intake data are generated in this review. Biomarkers: DHA status is described using plasma, serum, and immune cell membrane phospholipid DHA content (monocytes, lymphocytes, neutrophils) from cited studies. No original biomarker measurements are reported.	DHA is consistently described as an immunomodulatory and anti-inflammatory fatty acid. Associated with reduced production of pro-inflammatory cytokines (e.g., TNF-α, IL-1*β*, IL-6). Modulates innate immune cell activity and influences adaptive immune responses (T- and B-cell function). Promotes pro-resolving inflammatory pathways via specialized lipid mediators. In older adults, DHA may contribute to attenuation of chronic low-grade inflammation, although effects are heterogeneous across studies.
Chamorro (78), 2020, Chile	High income	Cross-sectional observational study	Healthy young men aged 18–25 years; vegans (*n* = 34) and omnivore controls not consuming fish or seafood (*n* = 33); all participants with normal BMI.	Vegetarian dietary patterns	Biomarkers: Plasma DHA: Vegans 0.64 ± 0.26 mol% vs. controls 1.56 ± 0.40 mol% (*p* < 0.0001). Erythrocyte phospholipid DHA: Vegans 1.41 ± 0.6 mol% vs. controls 3.44 ± 1.2 mol% (*p* < 0.0001). Spermatozoa DHA: Vegans 1.43 ± 0.3 mol% vs. controls 4.63 ± 0.7 mol% (*p* = 0.004)	Vegans showed significantly lower DHA levels in plasma, erythrocytes, and spermatozoa compared with omnivore controls. Despite higher dietary ALA intake, tissue DHA levels remained markedly lower in vegans. DHA levels in spermatozoa were substantially reduced in vegans compared with controls.
Chamorro (77), 2023, Chile	High income	Cross-sectional study	Adult men (*n* = 140): vegans (*n* = 35), omnivores (*n* = 35), omnivores with fish intake (*n* = 35), and pescatarians (*n* = 35).	Vegetarian dietary patterns	Dietary intake: Higher α-linolenic acid (ALA) and total n-3 PUFA intake in vegan men compared with omnivores and pescatarians (*p* = 0.025). Plasma DHA: VEG: 0.78 ± 0.10 mol%; OMV-1: 1.53 ± 0.30 mol%; OMV-2: 4.92 ± 0.30 mol%; PESC: 5.27 ± 0.40 mol% (*p* = 0.01). Erythrocyte DHA: VEG: 1.79 ± 0.30 mol%; OMV-1: 3.12 ± 1.00 mol%; OMV-2: 4.26 ± 1.30 mol%; PESC: 4.02 ± 0.90 mol% (*p* = 0.001). Spermatozoa DHA: VEG: 1.37 ± 0.20 mol%; OMV-1: 4.71 ± 0.40 mol%; OMV-2: 5.39 ± 0.60 mol%; PESC: 5.27 ± 0.40 mol% (*p* = 0.017).	Comparative analysis: Vegan men had significantly lower DHA concentrations in plasma, erythrocytes, and spermatozoa compared with omnivores and pescatarians (*p* < 0.05). Dietary pattern: Despite higher intake of α-linolenic acid (ALA), vegans exhibited limited endogenous conversion to DHA. The authors highlight that microalgae-derived DHA supplementation or DHA-fortified foods may be necessary to maintain optimal DHA status in vegan populations.
Chang (50), 2021, Malaysia	Upper middle income	Randomized crossover trial	Healthy overweight adults (*n* = 49; 35% men; mean age 29 ± 7 y; BMI 23.0–27.4 kg/m^2^)	Dietary patterns	Dietary intake: DHA intake from salmon: median 0.06 g/day (IQR 0.20) after 8 weeks. DHA intake from yellowstripe scad (YSS): median 0.04 g/day (IQR 0.11) after 8 weeks.; No significant between-group difference in DHA intake (*p* = 0.24). Biomarkers: Serum DHA: No significant changes after either YSS or salmon intervention; no significant between-group difference.	Serum DHA concentrations did not change significantly after 8 weeks of fish consumption in either intervention period (salmon vs. yellowstripe scad), with no between-group differences (*p* > 0.05). Despite fish intake, DHA intake remained low (≈ 0.04–0.06 g/day) and was not sufficient to elicit measurable increases in circulating DHA. Improvements observed in selected cardiometabolic markers (e.g., lipid profile and inflammatory indicators) occurred without concurrent changes in serum DHA, indicating that DHA was not the primary driver of these effects within the intervention period.
Chen (62), 2025, United Kingdom	High income	Prospective cohort study	Adult men and women from the UK Biobank. Free of rheumatoid arthritis (RA) at baseline; *n* = 188,597 participants. Age at baseline: mean ≈ 56 years. Follow up: ~9 years	Immune system function	Dietary intake: Assessed using repeated 24-h dietary recalls (Oxford WebQ); DHA intake estimated from food composition databases (UK Nutrient Databank); DHA analyzed as per 1-SD increment in intake. Biomarkers: Plasma DHA concentration measured at baseline in a subset using NMR-based metabolomics (Nightingale platform); Plasma DHA analyzed as a continuous variable (per SD increment).	Dietary DHA intake: Higher DHA intake was associated with a lower risk of incident RA. Per 1-SD increase in DHA intake: HR 0.90 (95% CI 0.85–0.95). Plasma DHA: Higher circulating DHA was associated with a lower risk of incident RA. Per 1-SD increase in plasma DHA: HR 0.83 (95% CI 0.74–0.93). Genetic risk interaction: The inverse association between DHA intake and RA was observed only among individuals with high genetic risk for RA. No significant association in those with low genetic risk. Overall pattern: Associations were linear and inverse, consistent for dietary intake and biomarker-based DHA.
Coniglio (34), 2023, Multiple countries	High income Middle income countries	Narrative review	Not applicable (review; no single study population)	Immune system function	No quantitative dietary DHA intake data reported. No primary DHA biomarker measurements reported; the review summarizes prior evidence on DHA incorporation into cell membranes and phospholipids.	DHA is described as a key modulator of immune and inflammatory responses, acting through suppression of pro-inflammatory signaling pathways (e.g., NF-κB) and through the generation of DHA-derived specialized pro-resolving mediators (resolvins, protectins, neuroprotectins). Incorporation of DHA into cell membrane phospholipids is reported to modify membrane microdomain organization, with downstream effects on immune cell activation and signaling. DHA is described as contributing to the resolution phase of inflammation, including effects on innate immune cells and, in the central nervous system, modulation of microglial inflammatory responses
Forsyth, 2016 (11), Multiple countries	Multiple income levels	Ecological study	General populations across multiple countries and regions worldwide; adults and general population dietary data derived from national surveys and food balance data	Dietary patterns	Dietary intake (primary focus): Mean adult DHA intake varied widely across countries, ranging approximately from <50 mg/day in many low- and middle-income countries to >200–300 mg/day in high-income countries with high fish consumption. Only a minority of countries achieved mean intakes consistent with commonly cited recommendations (≈ 200 mg/day DHA). DHA intake was strongly driven by marine food consumption; vegetarian or low-fish dietary patterns resulted in near-zero DHA intake.	High-income countries: Mean adult DHA intakes are commonly reported in the range of ≈200–400 mg/day, with multiple countries meeting or exceeding the ≈200 mg/day intake level, largely due to regular fish and seafood consumption. Upper-middle-income countries: Mean DHA intake is moderate and heterogeneous, generally ≈50–200 mg/day, reflecting variability in seafood availability and dietary patterns; only a limited number of countries reach recommended intake levels. Lower-middle- and low-income countries: Mean DHA intake is consistently low, typically <50 mg/day and frequently <10–20 mg/day, with some populations exhibiting near-zero intake due to minimal marine food consumption. A marked gradient in DHA intake is observed across national income levels, with progressively lower intakes as country income decreases, indicating that inadequate DHA intake is predominantly concentrated in low- and middle-income settings.
Gogga (74), 2024, Poland	High income	Cross-sectional study	Healthy adult women (*n* = 102), divided into four dietary groups: vegans (*n* = 30), vegetarians (*n* = 24), pescatarians (*n* = 12), and omnivores (*n* = 27)	Vegetarian dietary patterns/Immune system function (inflammation)	Dietary intake (DHA): Not reported separately. Dietary data report EPA + DHA combined: Vegans: 0.00 ± 0.00 g/day; Vegetarians: 0.02 ± 0.04 g/day; Pescatarians: 0.03 ± 0.43 g/day; Omnivores: 0.10 ± 0.19 g/day. Biomarkers (serum DHA, % of total fatty acids) (Table 2): Vegans: 0.22 ± 0.17; vegetarians: 0.48 ± 0.27; Pescatarians: 0.30 ± 0.10; Omnivores: 0.34 ± 0.12	Serum DHA concentrations varied by dietary pattern, with lower values in vegans compared with all other groups. Vegetarians exhibited higher serum DHA levels compared with pescatarians and omnivores. Differences in serum DHA across dietary groups were not accompanied by higher systemic inflammation, as CRP concentrations were similar across groups, with no evidence of elevated CRP in groups with lower DHA status.
Guadarrama-Lopez (75), 2015, Mexico	Upper middle income	Cross-sectional study	Adults (*n* = 240): 120 with T2M and 120 without T2M; 25–60 y; State of Mexico.	Immune system function	Mean habitual intake (g/day): n-3 PUFA = 0.68 ± 0.55 (T2D) vs. 0.81 ± 0.53 (non-T2D), *p* = 0.001. DHA = 0.09 ± 0.15 (T2D) vs. 0.13 ± 0.34 (non-T2D), not significant. n-6 PUFA = 4.1 ± 3.0 (T2D) vs. 5.3 ± 3.5 (non-T2D), *p* = 0.001.	Biochemical outcomes: Triacylglycerols (mg/dL): 256.8 ± 183 vs. 179.6 ± 39.5; *p* = 0.001. IL-6 (pg/mL): 18.3 ± 11.7 vs. 3.7 ± 0.12; *p* = 0.018. Correlations (T2D group): DHA vs. TNF-α *r* = 0.404 (*p* = 0.001); EPA vs. TNF-α *r* = 0.284 (*p* = 0.002). Lower n-3 PUFA intake and higher IL-6/TNF-α levels in T2D; positive correlations between DHA intake and inflammatory markers.
Hansson (70), 2015, United States	High income	Cross-sectional study	Healthy women, 18–47 years (mean age 26.1 ± 6.2 years)	Immune system function	Dietary intake: Habitual fatty fish intake assessed by FFQ and categorized into quartiles (<88, 88–174, 175–275, >275 g/week). Biomarkers: Serum phospholipid DHA measured by GC–MS. Mean serum DHA concentration: 0.056 ± 0.020 mg/mL. Serum DHA concentrations increased significantly across fatty fish intake quartiles (*p* = 0.002).	Serum DHA and oxidative stress: Serum DHA positively correlated with fish intake (*p* = 0.002). Fatty fish intake >275 g/week vs. < 88 g/week: 64% higher serum DHA (*p* < 0.001). DHA inversely associated with urinary 8-iso-PGF2α (oxidative stress marker): *p* = 0.049. Fatty fish intake inversely associated with urinary 8-iso-PGF2α: *p* = 0.01. Higher fish intake increased serum DHA and reduced oxidative stress markers.
Shu (64), 2025, United States	High income	Prospective cohort study (PLCO Cancer Screening Trial dietary questionnaire cohort)	Men aged 55–74 years from a U.S. screening cohort (PLCO)	Cancer risk	Dietary intake: DHA intake estimated using a validated FFQ (PLCO DHQ), expressed in g/day and analyzed in quintiles. Biomarkers: Not reported.	Incident prostate cancer: No association across DHA intake quintiles (Q5 vs. Q1): HR = 1.00 (0.90–1.12). Prostate cancer mortality: No statistically significant association after multivariable adjustment (Q5 vs. Q1): HR = 0.83 (0.62–1.12)
Tian (69), 2023, United Kingdom (UK Biobank)	High income	Cross-sectional analysis with mediation models (UK Biobank)	Adults aged 40–69 years at baseline. Analytical sample: ≈20,000 participant	Liver disease	Dietary intake: Dietary intake was assessed using repeated 24-h dietary recalls. DHA intake was not used as an isolated exposure in the main models; instead, DHA was considered within dietary patterns. Biomarkers: Serum DHA (% of total fatty acids) quantified and analyzed as a continuous variable. DHA was evaluated independently from other fatty acids and as a mediator of dietary pattern effects.	Outcome: Non-alcoholic fatty liver disease (NAFLD), defined by MRI-proton density fat fraction (MRI-PDFF ≥ 5%). Direct association: Higher serum DHA was inversely associated with NAFLD prevalence: OR = 0.70 (95% CI: 0.59–0.82) after multivariable adjustment. Mediation analysis: DHA partially mediated the inverse association between a PUFA-enriched vegetarian dietary pattern and NAFLD: Proportion mediated by DHA: ~10% of the total protective effect (*p* < 0.001).

GNI, Gross National Income; DHA, Docosahexaenoic Acid; EPA, Eicosapentaenoic Acid; ARA, Arachidonic Acid; PUFA, Polyunsaturated Fatty Acid; LC n-3 PUFA, Long-Chain Omega-3 Polyunsaturated Fatty Acids; FFQ, Food Frequency Questionnaire; RBC, Red Blood Cell; HR, Hazard Ratio; OR, Odds Ratio; CI, Confidence Interval; LDL, Low-Density Lipoprotein; HDL, High-Density Lipoprotein; VLDL, Very Low-Density Lipoprotein; BP, Blood Pressure; T2D, Type 2 Diabetes; IL, Interleukin; TNF-α, Tumor Necrosis Factor-alpha; CRP, C-Reactive Protein; PAI-1, Plasminogen Activator Inhibitor-1; ALA, Alpha-Linolenic Acid; MUFA, Monounsaturated Fatty Acid; SFA, Saturated Fatty Acid; NAFLD, Non-Alcoholic Fatty Liver Disease; IHD, Ischemic Heart Disease; RCT, Randomized Controlled Trial; LC–MS/MS, Liquid Chromatography–Tandem Mass Spectrometry; GC–MS, Gas Chromatography–Mass Spectrometry; MRI-PDFF, Magnetic Resonance Imaging–Proton Density Fat Fraction; NHANES, National Health and Nutrition Examination Survey; UK Biobank, United Kingdom Biobank; REGARDS, Reasons for Geographic and Racial Differences in Stroke; PLCO, Prostate, Lung, Colorectal, and Ovarian Cancer Screening Trial.

GNI classification follows the World Bank Atlas method for the 2026 fiscal year, based on 2024 GNI per capita.

DHA exposure was assessed using dietary intake estimates, biochemical biomarkers (e.g., plasma, erythrocytes, adipose tissue fatty acid composition), or other biological samples, as reported in each study.

Reported outcomes reflect associations between DHA intake or status and health-related indicators across multiple domains; causality should not be inferred unless supported by randomized controlled trial designs.

Methods used to assess DHA exposure and health outcomes varied across studies; therefore, direct quantitative comparisons should be interpreted with caution.

For narrative reviews, systematic reviews, and meta-analyses not restricted to a single country, national income classification reflects the predominant income level of the countries from which the evidence originates and is used for organizational purposes.

Adult and older adult populations include heterogeneous age ranges and health conditions, as defined in the original studies.

### 3.1 Dietary DHA intake and cross-country disparities by income classification

The primary source of essential omega-3 polyunsaturated fatty acids comes from the fat found in fish and seafood. The levels of DHA vary depending on the fish species, season, physiological state of the fish, and the region where they are caught. For example, fish from southern waters contain more DHA. Additionally, certain freshwater fish, particularly salmon, are rich in omega-3 fatty acids. Other sources include various seafood, such as oysters, shrimp, and crabs, as well as algae (48). However, studies have shown that reaching a daily intake of 2 g of DHA would require large dietary doses, sometimes even necessitating supplementation (41). While marine sources remain the primary contributors of DHA, plant-based diets and vegetarian patterns provide little to no preformed DHA, relying instead on the inefficient conversion of ALA to DHA (1). These considerations underscore the need to examine DHA intake within a broader socioeconomic framework, particularly when comparing populations across countries with different income levels.

Disparities in DHA intake across countries are shaped by multiple factors, including dietary patterns, food availability, and economic conditions. Stratification by national income level, as defined by the World Bank (81), provides a descriptive approach to examine how socioeconomic context may influence access to DHA-rich foods and subsequent intake levels. However, the available evidence assessing DHA intake across income groups remains limited and heterogeneous. Among the studies included in this review, only one ecological study evaluated DHA intake at a global level, using Food and Agriculture Organization (FAO) food balance sheets rather than nationally representative dietary intake surveys (11). No studies were identified that reported comprehensive national dietary intake data for DHA in adults across countries, limiting the ability to directly assess whether population-level intake meets existing recommendations by income classification. Of the eight studies conducted in UMIC (44, 50, 67, 68, 72, 75, 76, 80), none provided nationally representative intake estimates; instead, all were restricted to specific population subgroups, further complicating cross-country and cross-income comparisons. Importantly, no eligible studies were identified that evaluated dietary DHA intake in adults from LIC or LMIC. Taken together, these findings highlight substantial evidence gaps in the assessment of DHA intake across income levels and underscore pronounced inequities in the availability of intake data, particularly in lower-income settings. This lack of population-level evidence limits the capacity to evaluate global adherence to DHA-related recommendations and represents a critical barrier to understanding nutrition-related disparities across economic contexts.

In high-income settings, regular consumption of marine-derived foods and, in some cases, the use of supplements are more common, whereas in lower-income contexts, limited availability, affordability, and food system infrastructure may constrain dietary DHA intake (82). As a result, population-level DHA status is expected to vary substantially across countries according to socioeconomic conditions (11). Consistent with this pattern, the available empirical evidence on dietary DHA intake and biochemical status in adults is largely derived from HIC, including those in Europe and Latin America such as Sweden, Spain, Poland, and Chile, several studies have assessed the relationship between fish consumption, dietary patterns, and DHA status. For example, a cross-sectional study in Sweden (HIC) including 81 adults reported a positive correlation between serum DHA concentrations and fish intake, with participants consuming more than 275 g of fish per week exhibiting 64% higher serum DHA levels compared with those consuming less than 88 g per week (*p* < 0.001) (70). Similarly, in 2014, an RCT was conducted in Spain with 257 participants who underwent a 16-week intervention, 8 weeks with fish and 8 weeks without fish. There was a significant rising effect of the intervention with fish (seven servings of hake per week vs. no fish or seafood) on serum DHA (treatment effect *p* < 0.001, period effect *p* = 0.80, carryover effect *p* = 0.43).

Furthermore, the concentration of DHA has been evaluated based on the type of diet followed, as some authors report that vegans may have 0–40% lower plasma DHA concentrations compared to omnivores (39). To assess this, in Poland (HIC), a one-week follow-up was conducted with 71 vegans/pescatarians and 31 omnivores. The group with the lowest DHA concentration was the vegans, with 0.22%, followed by the vegetarians with 0.48%, the omnivores with 0.63%, and the group with the highest DHA concentration was the pescatarians with 0.74%. It is important to note that the pescatarians showed a significant difference only when compared to the vegans and vegetarians (*p* < 0.001) (74). In Chile, a HIC, a cross-sectional study was conducted evaluating 140 individuals, including omnivores with and without fish and seafood consumption, pescatarians, and strict vegans. The study found that neither the vegan group nor the control group consumed DHA. However, vegans had, on average, 0.92 % mol lower plasma DHA concentration compared to the other groups (0.64 ± 0.26 mol% vs. 1.56 ± 0.40 mol%). Additionally, a 2.03% lower erythrocyte phospholipid DHA concentration was observed in vegans (1.41 ± 0.6 mol% vs. 3.44 ± 1.2 mol%), as well as a 3.2% lower DHA concentration in spermatozoa (1.43 ± 0.3 mol% vs. 4.63 ± 0.7 mol%), all showing statistically significant differences (*p* < 0.01) (77). Overall, findings from high-income countries show that DHA status differs across dietary patterns and levels of fish consumption, highlighting the potential role of food availability and dietary context in shaping cross-country differences.

Compared with HICs, data from middle-income regions remain scarce and heterogeneous, reflecting gaps in population-level surveillance of DHA intake. In MIC, dietary intake has been investigated; however, available data are not nationally representative, as most estimates come from small, non-probabilistic samples rather than population-based nutrition surveys. Consequently, these findings should be interpreted with caution, as they may not be generalizable to the broader population. A cross-sectional study conducted with 158 Guatemalan, an UMIC, women reported that their average DHA intake was 3.09 g/day ± 0.71 (80). In Mexico (UMIC), only two studies have reported DHA consumption. A cross-sectional study found that DHA intake was 0.09 g/day (95% CI: 0.06–0.13) among 36 Mexican participants (76). Additionally, in a cohort study also conducted in Mexico, DHA intake was significantly lower in individuals with type 2 diabetes compared with those without the disease (T2D: 0.68 ± 0.55 g/day vs. non-T2D: 0.81 ± 0.53 g/day; *p* = 0.001) (75). Evidence from high-income settings indicates that even moderate increases in fish consumption led to substantial improvements in DHA levels, whereas in middle-income countries, available data show greater variability and limited intake consistency.

**Figure 1:**
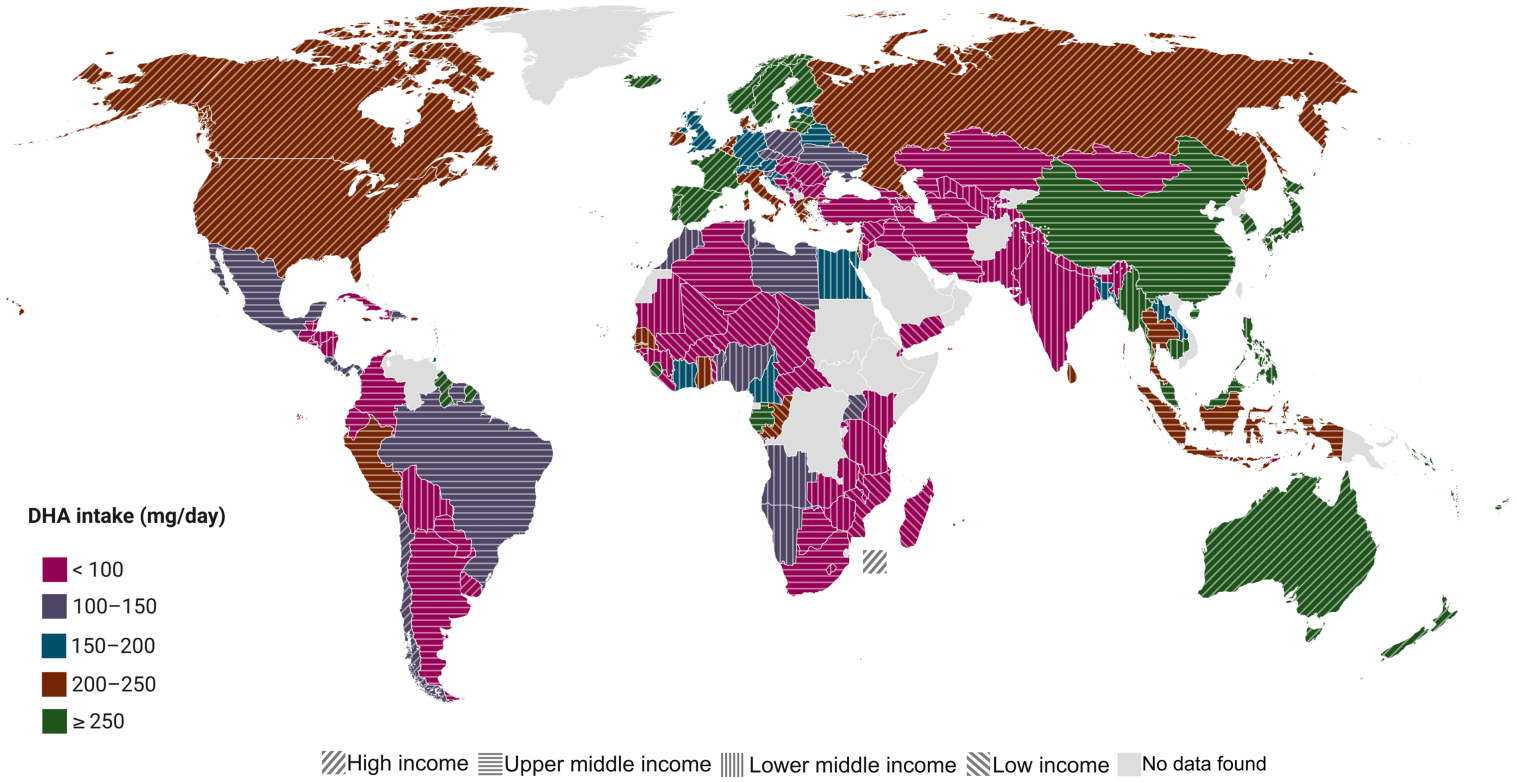
Global estimates of adult DHA intake (mg/day) according to World Bank gross national income (GNI) classification. Global estimates of DHA intake, according to Forsyth et al. (11), derived from the Food and Agriculture Organization of the United Nations (FAO) food balance sheets. These are presented by country and stratified by intake range (<100, 100–150, 150–200, 200–250, ≥250 mg/day; color-coded). Cross-hatching indicates GNI categories (high, upper-middle, lower-middle, and low income) according to the World Bank classification. The geographical distribution shows a consistent socioeconomic gradient: most LICs, LMICs, and UMICs have intakes below 150 mg/day, while most HICs exceed 200 mg/day. However, important discrepancy is also observed within each income group, indicating that the state of national dietary intake is not exclusively determined by economic capacity but is also influenced by food system governance, dietary habits, and cultural dependence on marine resources. Countries with limited coastlines or restricted access to seafood tend to be concentrated in the lowest consumption categories, while coastal and island nations exhibit greater availability even at similar income levels. The presence of large gray areas, denoting the absence of national estimates, highlights the lack of standardized dietary monitoring, national representative data, and control mechanisms, particularly in low- and middle-income regions. The figure was created using the Datawrapper platform (https://www.datawrapper.de/).

At the global level, disparities in DHA availability among adults have been examined primarily through ecological and modeling approaches rather than through nationally representative dietary intake surveys. To date, only one study has provided global estimates of DHA availability in adults using Food and Agriculture Organization (FAO) food balance sheets (FBS), which reflect national food supply rather than individual-level intake. In this analysis, Forsyth et al. (11) estimated per-capita DHA availability across 172 countries and reported marked differences by national income level. Mean DHA availability was highest in HIC, averaging approximately 327 mg/day, followed by UMIC at around 150 mg/day, LMIC at approximately 90 mg/day, and LIC, where estimated availability was below 50 mg/day. These differences are visually reflected in Figure 1, which illustrates a clear income-related gradient, with a substantial proportion of LICs, LMICs, and several UMICs falling well below an estimated intake of 200 mg/day, a value frequently cited in the literature as a benchmark associated with potential cognitive, cardiovascular, and inflammatory benefits in adults (5, 8, 83). Importantly, the authors also highlighted considerable heterogeneity within income groups, particularly among UMICs, where estimated DHA availability ranged widely depending on regional dietary patterns, access to marine foods, and reliance on plant-based diets.

Recent comprehensive reviews further contextualize these disparities by highlighting structural limitations in the available evidence base. Lewis et al. (45) emphasizes that most data on DHA intake in adults originate from high-income countries and are frequently derived from cohort studies, convenience samples, or biomarker-based assessments rather than nationally representative dietary surveys. As a result, current knowledge on adult DHA intake in middle-income settings relies largely on fragmented evidence from specific subpopulations, limiting cross-country comparability and precluding robust evaluation of intake adequacy at the population level. Similarly, Calder et al. (44) notes that formal dietary recommendations for DHA or long-chain omega-3 fatty acids are unevenly distributed across countries, with clearer guidance and implementation frameworks predominantly established in high-income regions. In several middle-income countries, the absence of specific intake recommendations, coupled with structural barriers to accessing marine foods, further complicates the assessment and interpretation of observed intake patterns. Importantly, Okai-Mensah et al. (47) frame these intake disparities within a broader nutrition transition perspective, highlighting how economic constraints, food system inequities, and limited dietary diversity in several LIC, LMIC and UMIC restrict access to nutrient-dense foods, including sources of long-chain omega-3 fatty acids. Rather than providing intake estimates, this review underscores how structural and socioeconomic factors shape dietary patterns across income settings and contribute to persistent micronutrient gaps in adult populations.

**Figure 2:**
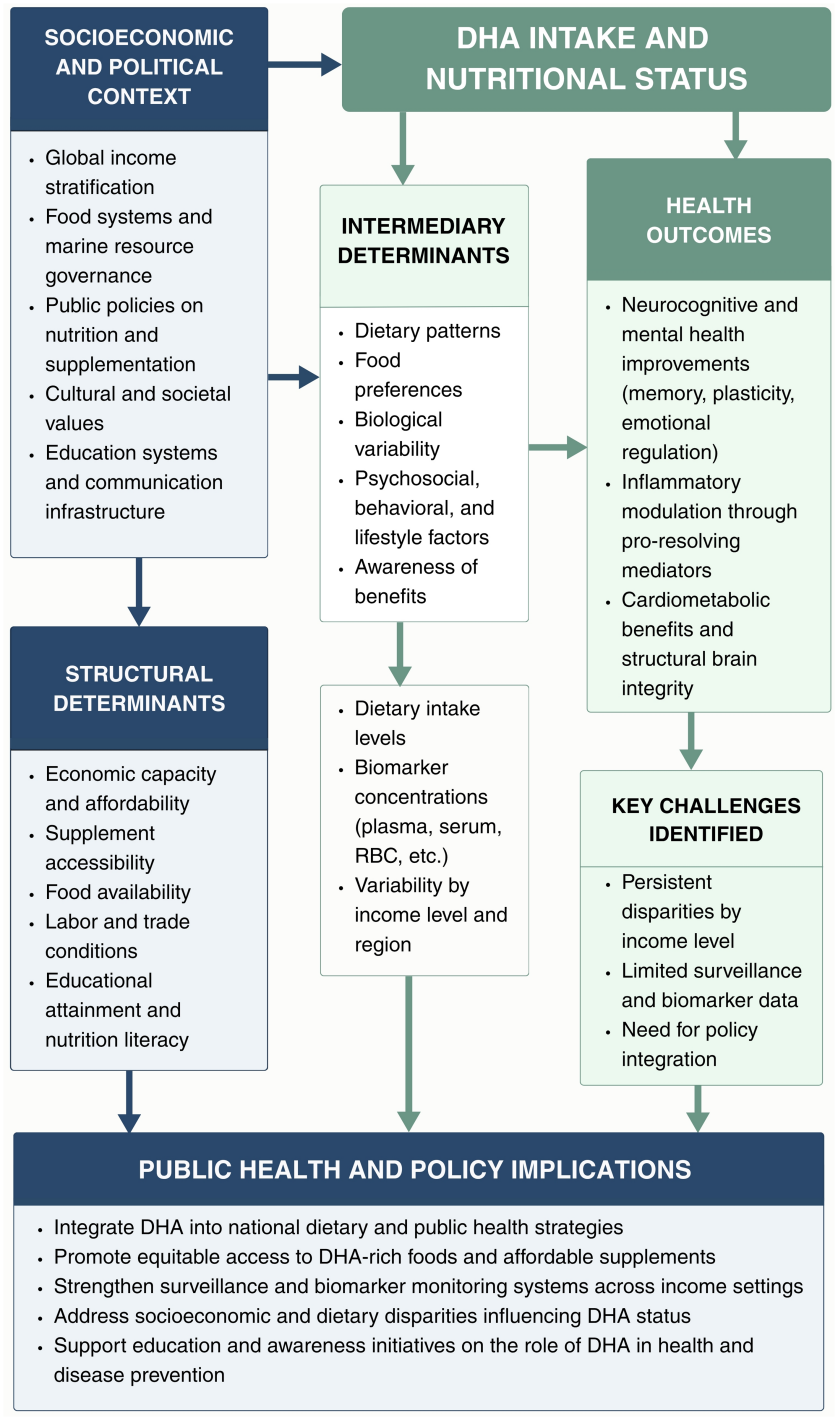
Conceptual framework illustrating the socioeconomic, structural, and intermediary determinants of DHA intake and associated health outcomes in adults. This framework describes the multilevel determinants that potentially influence DHA intake, nutritional status, and related health outcomes in adult populations. At the macro level, socioeconomic and political factors, such as income distribution, food system governance, and nutrition policies, establish the structural environment that regulates food affordability, access to supplements, and nutrition education. These structural conditions interact with intermediate determinants, including dietary patterns, food preferences, biological variability, and psychosocial or behavioral factors such as lifestyle, which together constitute DHA intake and biomarker concentrations at the individual and population levels. Differences in these determinants contribute to substantial variability in DHA status across regions and income levels within countries, with subsequent impacts on neurocognitive, inflammatory, and cardiometabolic outcomes. This framework also identifies persistent inequities and a lack of representative data at the national level, country surveillance systems, as one of the main critical barriers to assessing DHA adequacy, underscoring the need for coordinated public health strategies and policies aimed at promoting equitable access, fortification or supplementation, and strengthening biomarker monitoring and improving nutritional literacy globally.

These patterns are consistent with the conceptual framework presented in Figure 2, which highlights the interaction between structural determinants, such as food system organization, availability of marine foods, and economic capacity, and intermediary factors including dietary patterns, cultural preferences, and access to supplementation. In lower-income contexts, limited availability and affordability of DHA-rich foods may constrain population-level supply, whereas in higher-income countries, greater access to marine foods and supplements is more common. However, the wide intake ranges observed within income strata underscore the influence of country-specific factors beyond income classification alone. Importantly, because these estimates are based on food supply data rather than measured dietary intake or biomarkers, they also underscore the persistent lack of nationally representative intake data and biomarker surveillance in adults, particularly in low- and middle-income countries, limiting the ability to directly assess whether recommended intake levels are met across income settings. Building on the observed disparities in dietary DHA availability and intake across income settings, the following sections examine health outcomes associated with DHA in adults and older adults, with particular attention to how the distribution of evidence varies by national income classification. Rather than assuming uniform effects across contexts, this synthesis highlights where evidence is concentrated, where it is limited, and how structural and socioeconomic differences may shape both exposure to DHA and the outcomes evaluated. By organizing findings across specific health domains, these sections aim to contextualize reported associations within existing inequities in data availability, dietary patterns, and access to DHA-rich foods and interventions.

### 3.2 DHA and cognitive outcomes

Across the body of evidence identified in this review, cognitive and mental health-related outcomes associated with DHA were examined in 19 studies (33, 35–37, 40, 42, 45, 46, 48, 52, 54, 55, 57, 60, 67, 72, 73, 76, 79). Of these, the majority were conducted in HIC, while only three studies originated from UMIC, and none were identified from low- or lower-middle-income countries. This uneven distribution highlights an important evidence gap when considering potential disparities in neurocognitive outcomes related to DHA intake across income levels.

#### 3.2.1 DHA in brain structure and functions

**Figure 3:**
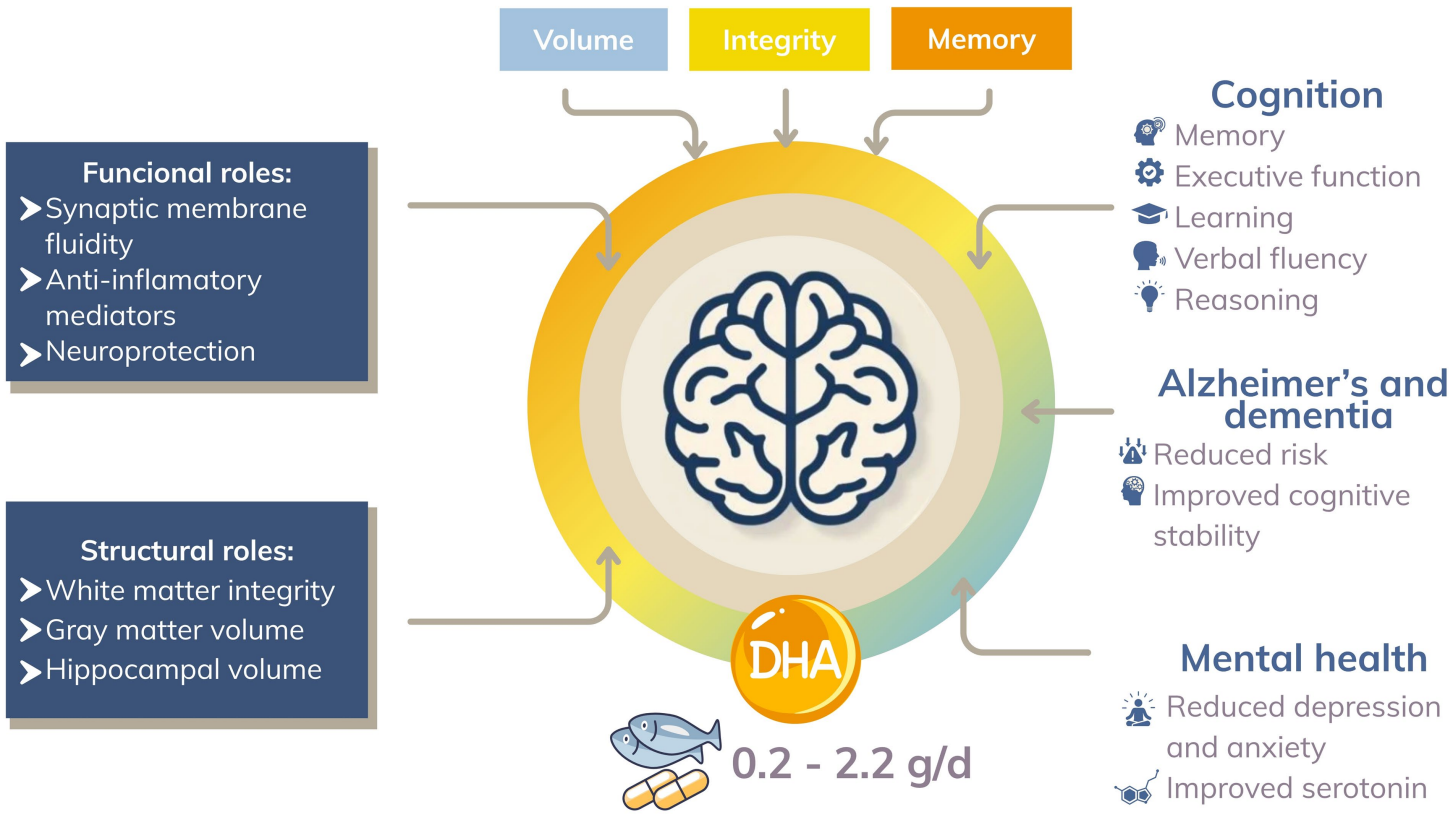
Functional and structural roles of docosahexaenoic acid (DHA) in adult brain integrity and cognitive health. This figure illustrates the main structural and functional pathways through which DHA supports brain volume, integrity, and cognitive performance in adults. Structurally, DHA is an essential component of neuronal membranes, contributing to the integrity of white and gray matter and preserving hippocampal volume, fundamental mechanisms for synaptic plasticity and neurogenesis. Functionally, DHA improves membrane fluidity, modulates the synthesis of anti-inflammatory lipid mediators, and exerts neuroprotective effects that maintain neuronal signaling and reduce oxidative stress. These processes result in improved memory, executive function, learning, and reasoning ability, while mitigating cognitive decline and the progression of neurodegenerative disorders such as Alzheimer’s disease. Furthermore, adequate DHA levels, usually ≥ 200 mg/day (5), have been associated with better mental health due to increased serotonergic activity and reduced systemic inflammation, contributing to a lower likelihood of depression and anxiety.

DHA is essential for brain function, as it is a part of its structure and participates in cognitive processes. Its role in neurological health has been widely studied. However, despite some favorable results, the absorption of DHA in the brain is limited, which could reduce its benefits for the brain and its functions. A narrative review conducted by Bazinet et al. (36), including interventions in high- HICs reported that DHA administration, through supplementation or intravenous delivery, demonstrated neuroprotective effects in preclinical models of cerebral infarction, spinal cord injury, and neuroinflammation. However, its therapeutic efficacy is limited by the slow absorption into the brain. Steady-state infusion studies have suggested that DHA synthesis could even exceed the brain uptake requirements. DHA uptake in the brain is estimated to be around 2.4 to 3.8 mg/day, with the main source being non-esterified plasma DHA. Although it represents only 5% of circulating DHA, it plays a key role in brain function (39). To explore how DHA is absorbed in the brain, a six-month supplementation was conducted, finding that the levels in cerebrospinal fluid increased by 28%, while plasma levels rose by 200%, suggesting that DHA transport to the brain is restricted (33). These findings suggest that inadequate DHA intake or chronically low consumption may have disproportionate implications for brain structure and function, particularly in populations with limited dietary access to DHA-rich foods. The underlying mechanisms governing DHA transport, turnover, and structural roles in the brain, along with their implications for neuroprotection and cognition, are summarized in Figure 3.

DHA has been extensively studied for its role in the structural integrity of the brain, including its relationship with brain volume, white matter, and gray matter. A narrative review by Macaron et al. (42), including magnetic resonance imaging studies conducted in a HIC settings, identified a positive correlation between DHA concentrations and hippocampal volume, a critical region for memory processing. In the same review, findings from a cross-sectional study with 1,575 cognitively healthy participants of the Framingham Study showed that individuals in the highest quartile of red blood cell DHA had significantly larger total brain volumes compared with those in the lowest quartile, independent of APOE ε4 genotype, homocysteine levels, and other vascular risk factor; however, authors found no evidence that DHA had protective effects against atrophy in the hippocampus or right amygdala. Similarly, in a cross-sectional study conducted among 40 cognitively healthy older adults from the Adventist Health Study-2 in the United States (HIC), DHA and EPA were associated with greater white matter volume (r = 0.33, *p* < 0.05), although no associations were observed with hippocampal volume or frontal pole cortical thickness (73). Evidence also indicates that DHA contributes to the microstructural integrity of white matter, which is essential for maintaining neuronal connectivity. In contrast, in a RCT included in a HIC, included narrative review, involving 65 participants reported that supplementation with 2.2 g/day of DHA for 26 weeks increased left hippocampal gray matter volume, suggesting potential structural benefits of DHA in regions critical for memory processing (41). Taken together, the predominance of evidence from HIC constrains inference regarding DHA-related brain structural outcomes in lower-income populations and highlights the need for context-specific data to evaluate whether socioeconomic disparities contribute to differential neurocognitive risk associated with inadequate DHA intake.

#### 3.2.2 DHA in cognitive performance

Beyond its structural role, DHA contributes to neuronal function through its involvement in synaptic membrane integrity, neuroprotection, and the regulation of inflammatory signaling pathways. Evidence supporting these mechanisms is largely derived from studies conducted in high-income countries, where DHA released from membrane phospholipids by phospholipase A₂ is converted into bioactive lipid mediators, including neuroprotectin D1, resolvins, and maresins, which participate in neuronal survival, resolution of neuroinflammation, and brain repair processes under conditions such as injury or ischemia (36, 54). In addition, DHA modulates key neuronal signaling pathways by influencing protein kinase C activity and synaptic protein assembly, thereby supporting neurotransmission, synaptic plasticity, and cognitive processes such as memory and learning. These mechanistic insights, primarily characterized in high-income settings, also provide biological plausibility for the associations observed between low DHA status and neurodevelopmental or neurodegenerative conditions (36, 48). However, whether these mechanisms operate similarly in populations from middle- and low-income countries remains largely unexplored.

The influence of DHA on cognitive performance has been widely investigated across observational and interventional studies. A narrative review conducted in 2021 reported that supplementation with 2.2 g/d of DHA for 26 weeks improved executive function in an RCT with 65 participants from a HIC (42). Similarly, another review including most studies from a HIC (41), described consistent improvements in cognitive function associated with DHA. The authors also included observational studies that found that higher levels of DHA in serum phospholipids were associated with better performance in non-verbal reasoning task. In addition, authors reported an RCT involving 485 adults over 55 years of age found that supplementation with 0.9 g/day of algae-derived DHA for 6 months significantly reduced paired associative learning errors compared with placebo. Another trial with 65 individuals aged 50–75 years reported improvements in verbal fluency after DHA supplementation (36). Despite these findings, several observational studies have reported contrasting results. For instance, a cohort study conducted in the United States, a HIC, found no significant associations between plasma DHA concentrations and either cognitive decline or incident cognitive impairment among adults over 65 years of age (54). Similarly, a cross-sectional study conducted in a HIC, reported no correlations between DHA levels and measures of processing speed or executive function, regardless of age, sex, or educational level (73). Such inconsistencies could be explained by variations in study design, DHA dosage, intervention duration, and assessment methodologies.

Furthermore, memory-specific outcomes have been explored in a subset of observational and interventional studies, primarily through randomized controlled trials. Evidence summarized in narrative reviews from HIC, indicates that DHA supplementation has been associated with improvements in selected memory domains, including immediate and associative memory, in older adults receiving doses ranging from 0.2 to 2.2 g/day over intervention periods of three to twelve months (42). These benefits, however, have not been consistently replicated across studies. Several investigations conducted mostly in HIC, reported no significant associations between DHA exposure and memory performance, including immediate recall, attentional tasks, or performance on standardized cognitive tests such as the Stroop or digit span (73). Taken together, these findings suggest that while DHA supplementation may confer benefits for specific aspects of memory in certain populations, the evidence remains heterogeneous and insufficient to draw definitive conclusions regarding its effects across memory domains.

Collectively, evidence linking DHA intake or supplementation with cognitive performance in adults and older adults is largely restricted to HIC. The absence of comparable data from LIC, LMIC and UMIC, limits the ability to evaluate whether these cognitive effects are generalizable across income levels and highlights a critical gap in understanding potential neurocognitive disparities related to DHA availability and intake.

#### 3.2.3 DHA in Alzheimer’s disease and dementia

Numerous studies have examined the association between DHA status and the risk of Alzheimer’s disease and other forms of dementia. In a Japanese cohort (HIC) of 1,127 individuals followed for 20 years, higher DHA intake was associated with a 27% lower risk of developing Alzheimer’s disease (HR = 0.73, 95%CI: 0.57, 0.95; *p* = 0.018) (52). Similarly, in the multiethnic U.S. WHICAP cohort (HIC) (*n* ≈ 2,612) followed for a mean of 4.5 years, higher dietary DHA intake was associated with a significantly lower risk of Alzheimer’s disease (HR = 0.73, 95% CI: 0.57–0.95; *p* = 0.018) (41). In a cohort study conducted with 129 Taiwanese patients with Alzheimer’s (HIC), it was found that the individuals with lower DHA levels at the start of the study had a 13.1% increase in the risk of cognitive decline (OR = 1.13, 95%CI: 1.020, 1.254; *p* = 0.020). Participants with higher plasma DHA levels (5.2 ± 7.1 mg/mL) exhibited greater cognitive stability compared with those who experienced cognitive deterioration (1.8 ± 3.6 mg/mL) (57).

Nevertheless, studies conducted in HIC settings, have reported contrasting results, with no consistent evidence supporting a protective effect of DHA against Alzheimer’s disease. Reduced DHA concentrations have consistently been reported in individuals with Alzheimer’s disease (0.56 ± 0.41) compared to those without the disease (1.15 ± 0.888) (40). Specifically, a decrease in DHA content has also been observed in the phospholipids of the frontal cortex of Alzheimer’s patients, which could be the result of high omega-6 fatty acid consumption, aging effects on desaturases, and increased lipid peroxidation. Even post-mortem studies in Alzheimer’s patients have revealed lower levels of unesterified DHA in the hippocampus, as well as lower levels of DHA-derived anti-inflammatory compounds, suggesting a potential link between DHA depletion and disease progression (36). However, an epidemiological study that assessed DHA intake and its relation to the risk of Alzheimer’s, a 39% reduction in the risk of the disease was observed, although this association did not reach statistical significance (37). Likewise, in a U.S. cohort study of 3,564 Alzheimer’s disease patients carrying the APOE ε4 negative genotype, showed that supplementation with 2 g/day of DHA for 18 months had no significant benefits for dementia prevention or protection against mild cognitive impairment (54).

Even the effect of DHA has been assessed according to the disease stage. While DHA supplementation has demonstrated benefits in some studies, results remain inconsistent, specifically among individuals with advanced dementia or severe cognitive decline (33, 36). In a narrative review including predominantly studies from a HIC, the authors studied the effects of DHA on cognitive performance and reported that although there were slight improvements in memory in individuals with mild cognitive impairment, in patients with advanced Alzheimer’s disease, the effects were not significant (33). These findings suggest that while DHA may have a positive impact in the early stages of cognitive decline, its effectiveness appears to decrease as neurodegeneration progresses.

Overall, evidence examining the association between DHA status and Alzheimer’s disease or dementia is derived almost exclusively from HIC. Although several cohort and observational studies conducted in HICs suggest that higher dietary or circulating DHA levels may be associated with a lower risk of Alzheimer’s disease or slower cognitive decline, findings remain heterogeneous and stage-dependent. Importantly, the absence of comparable data from LIC, LMIC, and UMIC precludes meaningful assessment of whether these associations extend across income settings. This gap limits understanding of potential inequities in dementia risk related to DHA availability and highlights the need for longitudinal studies in underrepresented regions, particularly as populations age globally.

### 3.3 DHA and mental health outcomes

Due to its role in maintaining brain structure and function, DHA has also been investigated in relation to mental health outcomes. Evidence from a narrative review including mostly studies form HIC, indicates that a lower concentration of DHA has been linked to a decreased level of 5-hydroxyindolacetic acid (5-HIAA), the primary metabolite of serotonin, in the cerebrospinal fluid. Reduced levels of 5-HIAA have been observed in individuals who have attempted violent suicide, those experiencing a state of aggression, and those with weakened impulse control, particularly in individuals suffering from depression, schizophrenia, alcohol addiction, and adjustment disorder. The authors suggest that low DHA levels may impair serotonergic neurotransmission. Moreover, ecological data have shown that populations with low fish consumption tend to exhibit higher rates of depression (48).

In middle-income countries settings, the association between DHA and mental health has also been explored. In a case–control study conducted in Iran, an UMIC, with 90 cases (45 with depression, stress, or anxiety and 45 controls) exhibited significantly lower erythrocyte DHA concentrations (0.832 ± 0.507 % of total fatty acids) compared to the control group (1.148 ± 0.594 %; *p* = 0.008) (67); moreover, DHA intake was further associated with a 70% lower risk of stress and anxiety (OR = 0.306; *p* = 0.014), suggesting a potential protective role of this fatty acid in mental health outcomes. Similarly, a cross-sectional study conducted in Brazil (UMIC), with 1,228 adults reported that individuals with anxiety consumed less DHA (0.42 g/day; 95%CI: 0.14, 0.79) than those without anxiety (0.46 g/day; 95% CI: 0.20, 0.88; *p* < 0.0001). Higher DHA intake was associated with an 18% reduction in anxiety risk (OR = 0.82; 95%CI: 0.69, 0.98) (72). This evidence indicates that DHA deficiency may contribute to the pathophysiology of mental health disorders, whereas adequate intake may help reduce stress, anxiety, and depression risk. However, further research is needed to confirm its therapeutic potential.

Evidence linking DHA status to mental health outcomes is derived predominantly from HIC, with a limited but emerging body of data from upper-middle-income settings. While studies conducted in HICs suggest plausible neurobiological mechanisms connecting low DHA levels with altered serotonergic function and adverse mental health profiles, evidence from UMICs provides complementary population-level and case–control data indicating inverse associations between DHA intake or status and anxiety, stress, and depressive symptoms. The absence of studies from LIC and LMIC restricts the ability to evaluate whether these associations differ across income strata or reflect broader inequities in dietary DHA access. This gap underscores the need for well-designed epidemiological and interventional studies in underrepresented income settings to better characterize potential mental health disparities related to DHA availability and intake.

### 3.4 DHA and cardiovascular outcomes

Cardiovascular health has been one of the most frequently explored domains in relation to DHA intake in adults and older adults. Within the studies included in this review, 17 articles evaluated cardiovascular-related outcomes (30, 31, 35, 39, 44, 47, 49, 51, 57–59, 63, 66, 68, 71, 78, 80), predominantly conducted in HIC. Only four studies were carried out in UMIC, while no evidence was identified from low- or lower-middle-income settings. The effects of DHA intake on cardiovascular health and related mortality have been widely studied. Although no significant association between DHA consumption and cardiovascular mortality has been consistently demonstrated, several studies have reported possible trends.

In a U.S. cohort study (HIC), the results showed that individuals with higher DHA intake had a 5% lower risk of all-cause mortality compared to the group with lower intake. The mean DHA intake in this population was 0.019 g/day, representing a small fraction of total omega-3 PUFA intake, primarily derived from plant-based sources. Only 0.5% of the participants met the recommended threshold of 0.25 g/day for combined EPA and DHA intake (61). Similarly, in a cohort study of 2,043 U.S. adults (59), higher plasma DHA concentrations were associated with a lower incidence of ischemic stroke, and DHA consumption reduced the risk of this event by 12%. Additionally, DHA mediated the relationship between fish consumption and the risk of stroke. It was also observed that higher fish consumption was associated with increased DHA levels (*β* = 0.36, 95%CI: 0.20, 0.52; *p* < 0.0001). Despite these findings, fish consumption among participants was low, with an average intake of 1.40 g/day, and 30% of participants reported not consuming fish at all. The daily DHA intake among both men and women ranged between 0 and 6.14 g/day, with half of the participants consuming 1.4 g/day or less. In Denmark, another HIC, a large prospective cohort study of 55,338 adults followed for 13.5 years assessed ischemic stroke subtypes by DHA intake. Individuals in the highest DHA intake group had a 28% lower risk of large artery atherosclerosis (HR = 0.72; 95%CI: 0.53, 0.99; *p* = 0.043) but a 112% higher risk of cardioembolic stroke (HR = 2.12; 95%CI: 1.12, 3.69; *p* = 0.002) (58). In the other hand, a case–control study from a UMIC, with 886 Iranians, it was found that DHA consumption decreases the probability of developing ischemic heart disease by 2% (OR = 0.98; 95%CI: 0.97, 0.99). The authors also reported significant differences in DHA intake between cases and controls, among individuals without ischemic heart disease, the DHA intake was 141.9 mg/day, while those with ischemic heart disease had a lower DHA intake of 113.6 mg/day (68), suggesting that inadequate DHA intake may increase susceptibility to ischemic heart disease.

Dyslipidemia and lipid alterations have also been studied in relation to DHA intake. An RCT conducted in 26,034 Americans (HIC), LDL and HDL cholesterol concentration was evaluated based on particle size according to DHA intake from different types of fish and seafood. The results showed that DHA is associated with a higher concentration of large LDL and HDL cholesterol particles. This is relevant because large LDL particles are less prone to oxidation and are considered less harmful to cardiovascular health, while large HDL particles are usually more effective in removing cholesterol from the arteries (66). In UMIC like Mexico, a cohort study including 240 adults (120 with and 120 without T2D), DHA intake was low in both groups, with slightly higher values in non-T2D individuals (0.13 ± 0.34 g/day vs. 0.09 ± 0.15 g/day). The T2D group also exhibited higher triacylglycerol concentrations (256.8 ± 183 mg/dL vs. 179.6 ± 39.5 mg/dL; *p* = 0.001), suggesting the coexistence of hypertriglyceridemia and reduced DHA intake. Additionally, a significant positive correlation was observed between DHA intake and serum TNF-*α* levels (r = 0.404; *p* = 0.001), indicating a potential pro-inflammatory state associated with low n-3 consumption in this population (75).

The association between DHA and blood pressure has been examined predominantly HIC, with overall findings remaining inconsistent. A meta-analysis including eight prospective cohort studies, mostly conducted in HIC, evaluated 56,204 adults with 20,497 incident cases of elevated blood pressure over follow-up periods ranging from 3 to 20 years (31). Fish consumption was not significantly associated with the risk of elevated blood pressure (summary RR = 0.96; 95% CI: 0.81–1.14), whereas higher circulating concentrations of long-chain n-3 PUFA were inversely associated with this outcome (summary RR = 0.67; 95% CI: 0.55–0.83). Specifically, biomarker-based DHA showed a significant inverse association with elevated blood pressure (summary RR = 0.64; 95% CI: 0.45–0.89), while dietary long-chain n-3 PUFA intake was not significant (summary RR = 0.80; 95% CI: 0.58–1.10). These findings suggest that circulating DHA may better capture the relationship between DHA and blood pressure regulation than self-reported dietary intake (31). Evidence from intervention and observational studies, largely conducted in HIC populations, remains heterogeneous. A 2022 narrative review of double-blind, placebo-controlled trials administering 4 g/day of DHA reported mixed effects on blood pressure, with some trials showing no significant changes and others reporting reductions in systolic and diastolic blood pressure (35). Similarly, a cross-sectional study conducted among 157 French Polynesians (HIC) found that each 1% increase in erythrocyte DHA concentration was associated with a reduction in resting heart rate (*β* = −2.57 beats/min; *p* = 0.005) and a decrease in diastolic blood pressure (−1.96 mmHg; *p* = 0.05), alongside improvements in heart rate variability (71). Differences across studies may reflect variation in study design, background pharmacological treatments, comorbidities, and methodological approaches, including placebo selection, highlighting the need for further research to clarify the independent effects of DHA on blood pressure and cardiovascular regulation (35, 71).

In summary, evidence on DHA and cardiovascular outcomes in adults is largely derived from high-income countries, with only four studies from upper-middle-income settings and no data from low- or lower-middle-income countries. While studies in HIC suggest potential associations between DHA status and cardiovascular markers, including ischemic stroke risk, lipoprotein profiles, and blood pressure regulation, findings remain inconsistent and highly dependent on study design and exposure assessment. In UMIC, available data indicate low DHA intake alongside unfavorable cardiometabolic profiles, but the limited scope of these studies restricts broader inference. The pronounced imbalance in evidence across income levels highlights a substantial knowledge gap and limits the ability to evaluate cardiovascular disparities related to DHA intake at the global level.

### 3.5 DHA and inflammation-related chronic conditions

Research addressing the relationship between DHA and inflammation-related chronic conditions remains scarce and is predominantly derived from HIC. Among the studies included in this review, nine evaluated outcomes related to cancer and immune or inflammatory processes (32, 34, 44, 53, 62, 64, 65, 70, 75), with only one conducted in an UMIC setting and no evidence from LIC.

The role of DHA in chronic diseases beyond cardiovascular health has been increasingly investigated, particularly in cancer and inflammatory conditions. In women, DHA has been studied for its potential protective role against endometrial cancer, possibly through its anti-inflammatory properties; however, the findings are still inconclusive. In 2023, Brasky and colleagues published a meta-analysis including data from HIC, in which they studied the effects of DHA on endometrial cancer. The results showed that higher DHA consumption increases the risk of endometrial cancer by 9% (OR = 1.09, 95%CI: 1.01, 1.19; *p* = 0.042). After stratifying by BMI, they found that specifically in overweight women (BMI ≥ 25 kg/m^2^), the risk of endometrial cancer was 19% higher (OR = 1.19, 95%CI: 1.08, 1.32; *p* = 0.003), whereas in women with normal weight according to this marker, no significant risk was demonstrated (OR = 1.00, 95%CI: 0.87, 1.15; *p* = 0.778) (53). In addition, a 9-year follow-up was conducted in a cohort study in the United States with 263 women. The results showed that the group with the highest DHA consumption had a 66% higher risk of endometrial cancer compared to the group with the lowest consumption (HR Q5 vs. Q1 = 1.66, 95%CI: 1.09, 2.55; *p* = 0.036) (53, 65). One possible explanation suggested by the authors is that the benefits of DHA on endometrial cancer risk may be more evident in women with normal weight than in women with obesity; the pro-inflammatory environment and elevated estrogen levels might interfere with the anti-inflammatory benefits of DHA that would help reduce the risk of this type of cancer. Although DHA may reduce estrogen synthesis via cyclooxygenase-2 inhibition, further studies using precise biomarkers of DHA exposure and controlling for obesity-related hormonal and inflammatory pathways are needed to clarify these inconsistencies and determine the direction of the association.

DHA intake has also been associated with breast cancer risk reduction. A case-cohort study conducted in 2,491 Australian, a HIC, women showed that the group with the highest DHA consumption had a 47% lower risk of developing breast cancer compared to women with the lowest consumption (HR Q5vsQ1 = 0.53, 95%CI: 0.38, 0.75; *p* = 0.002) (65). The authors also evaluated breast cancer by molecular subtype, finding that DHA consumption reduces the risk of breast cancer by 16% (HR = 0.84, 95%CI: 0.73, 0.96; *p* = 0.01). However, no significant differences were found (*p* = 0.06) in the plasma phospholipid percentage, with 0.143 (0.10–0.21) in non-cases and 0.140 (0.09–0.18) in cases. Due to the notable differences between studies, lack of data on DHA supplementation, and incomplete adjustment for confounders, the findings highlight the need for further RCTs and mechanistic studies to clarify DHA’s role in breast and endometrial cancer, particularly in higher-risk populations.

Regarding inflammation, DHA has been shown to modulate immune responses by downregulating proinflammatory cytokines such as interleukins (IL-1, IL-2, IL-6), interferon-*γ* (IFN-γ), and tumor necrosis factor-*α* (TNF-α) (48). A review including studies from both high- and middle-income countries (sample sizes from 67 to 5,000 participants) reported that DHA supplementation ranging from 300 mg/day to 1.2 g/day for 6 weeks to 18 months reduced inflammatory markers. In one RCT, supplementation with 1 g/day of DHA for 12 weeks reduced C-reactive protein levels by 25% compared with placebo (*p* < 0.05). In another trial, 600 mg/day for 6 months decreased IL-6 and TNF-*α* levels in patients with chronic inflammation (34). Consistent with these results, in a UMIC, Guadarrama-López et al. conducted a cohort study in Mexico to compare the effect of DHA on inflammatory cytokines in individuals with and without T2D. The results showed a correlation between TNF-α and DHA intake (r = 0.404; *p* = 0.001). Additionally, the group with T2D had the lowest DHA intake and the highest IL-6 concentration compared to individuals without T2D (*p* = 0.018) (75). Additionally, evidence from a large prospective cohort conducted in the United Kingdom (HIC) suggests a protective association between DHA intake and rheumatoid arthritis (RA). In a cohort of 188,597 RA-free adults from the UK Biobank, followed for a median of 9.1 years, each one–standard deviation increase in dietary DHA intake was associated with a 10% lower risk of incident RA (HR = 0.90; 95% CI: 0.85–0.95). This inverse association was linear and more pronounced among individuals with high genetic susceptibility to RA. Additionally, circulating DHA concentrations were independently associated with reduced RA risk, supporting a potential role of DHA in modulating immune and inflammatory pathways involved in RA pathogenesis (62).

DHA intake has been linked to a reduced risk of developing non-alcoholic fatty liver disease (NAFLD, recently redefined as metabolic dysfunction–associated steatosis liver disease [MASLD], will be referred to as NAFLD in this review, as this was the terminology used in the studies included). In a cross-sectional study conducted in the United Kingdom (HIC), with a 4-year follow-up of 93,399 participants, it was reported that DHA reduced the likelihood of NAFLD by 30% (OR = 0.7, 95%CI: 0.59, 0.82). Furthermore, DHA accounted for 10% of the total effect of a low-fat, high PUFA diet, which was associated with an 18% reduction in the risk of NAFLD (OR = 0.82, 95%CI: 0.78, 0.87) (69). This suggests that the treatment of simple liver steatosis may benefit from DHA and potentially help in reducing the risk of NAFLD.

Overall, evidence linking DHA to inflammation-related chronic conditions, including cancer, immune-mediated disorders, and liver disease, is predominantly derived from high-income countries, with very limited representation from upper-middle-income settings and no data from low- or lower-middle-income countries. While findings from HIC suggest potential associations between DHA intake or status and reduced inflammatory markers, autoimmune disease risk, and selected cancer outcomes, results remain heterogeneous and, in some cases, contradictory. The scarcity of studies from non-HIC contexts, coupled with differences in study design, exposure assessment, and confounder control, limits the generalizability of these findings across income levels.

## 4 Conclusions and public health implications

Docosahexaenoic acid (DHA) is an essential long-chain omega-3 fatty acid with a central role in membrane integrity, neuronal function, and the modulation of inflammatory and cardiometabolic processes. This narrative review highlights pronounced disparities in both DHA exposure and the distribution of health-related evidence when findings are stratified by gross national income.

Among the studies included, most evidence originates from HIC, where adult populations generally exhibit higher dietary DHA intake and more favorable biochemical status, likely reflecting differences in dietary patterns, seafood availability, and access to supplementation. In UMIC, available evidence is limited in number and primarily derived from specific population subgroups rather than nationally representative samples. No eligible studies meeting the predefined inclusion criteria were identified from low-income or lower-middle-income countries (LIC/LMIC) in adult populations. This absence should not be interpreted as evidence that DHA intake or related outcomes have not been examined in these settings, but rather reflects limitations related to study design, population coverage, indexing, publication visibility, and eligibility criteria applied in this review.

The uneven distribution of evidence by income level has important implications for interpreting associations between DHA and health outcomes. While studies conducted in HIC report associations between DHA intake or status and cardiovascular, cognitive, mental health, and inflammation-related outcomes, findings remain heterogeneous and dependent on methodological factors, including exposure assessment, baseline intake, and population characteristics. Evidence from UMIC is comparatively sparse and inconsistent, and the lack of data from LIC/LMIC precludes assessment of whether observed associations are consistent across income settings or reflect context-specific patterns of exposure and risk.

Several factors may contribute to variability in reported findings, including differences in DHA dose, duration of exposure, dietary versus biomarker-based assessment methods, control for confounding variables, and background dietary patterns. In addition, structural determinants related to GNI, such as food system organization, affordability and availability of marine foods, and national nutrition policies, are likely to influence DHA exposure but are infrequently examined explicitly in existing studies.

From a public health perspective, these findings highlight the need to strengthen nutritional surveillance systems that incorporate both dietary intake data and biochemical indicators of DHA status, particularly in middle- and lower-income settings. Generating nationally representative data is necessary to evaluate population-level intake relative to existing recommendations and to identify potential gaps in exposure. Future research should prioritize well-designed epidemiological studies and randomized controlled trials in underrepresented income settings, as well as systematic reviews that formally assess risk of bias and regional underrepresentation in the literature.

Improving access to DHA-rich foods through context-appropriate dietary strategies, supplementation, or food fortification may contribute to reducing income-related disparities in cardiometabolic, neurocognitive, and inflammation-related health outcomes. Addressing current evidence gaps is essential for informing context-specific recommendations and supporting equitable nutrition policies across adulthood and aging.
