# Supplementary material for: Docosahexaenoic acid intake and health in adults and older adults: a narrative review of disparities by country income level
Source: Front Nutr. 2026 Mar 20;13:1742942. doi: 10.3389/fnut.2026.1742942 (PMC13046497; doi:10.3389/fnut.2026.1742942)
Supplement: Supplementary file 1 [file Table_1.docx]

Supplemental Material

# Supplementary Data

| **Supplemental Table 1.** Organization of DHA-Related Health Outcomes in Adults and Older Adults by Health Domain | | | | | | |  |
| --- | --- | --- | --- | --- | --- | --- | --- |
| **Outcome** | **Cardiovascular Disease** | **Cognition and Mental Health** | **Liver Disease** | **Cancer Risk** | **Immune System Function** | **Vegetarian Dietary Patterns** |  |
| Blood pressure regulation | ✓ | X | X | X | X | X |  |
| Heart rate variability | ✓ | X | X | X | X | X |  |
| Reduction in triglycerides | ✓ | X | ✓ | X | X | X |  |
| Cognitive decline prevention | X | ✓ | X | X | X | X |  |
| Mood and depression management | X | ✓ | X | X | X | X |  |
| Neuroprotection | X | ✓ | X | X | X | X |  |
| Liver fat reduction | X | X | ✓ | X | X | X |  |
| Liver inflammation control | X | X | ✓ | X | X | X |  |
| Lower risk of certain cancers | X | X | X | ✓ | X | X |  |
| Anti-inflammatory outcomes | ✓ | ✓ | ✓ | ✓ | ✓ | X |  |
| Immune function enhancement | X | X | X | X | ✓ | X |  |
| Reduced infection susceptibility | X | X | X | X | ✓ | X |  |
| DHA adequacy in vegetarian diets | X | X | X | X | X | ✓ |  |
| ✓ indicates inclusion in the domain X indicates exclusion.  This classification synthesizes the outcomes identified in the present study for organizational purposes, based on current scientific evidence on DHA in adult and older populations.  Anti-inflammatory properties refer to biomarkers or clinical indicators reported within each health domain (e.g., cytokines, CRP, or disease-specific inflammatory markers | | | | | | |  |
|  |  |  |  |  |  |  |  |

**
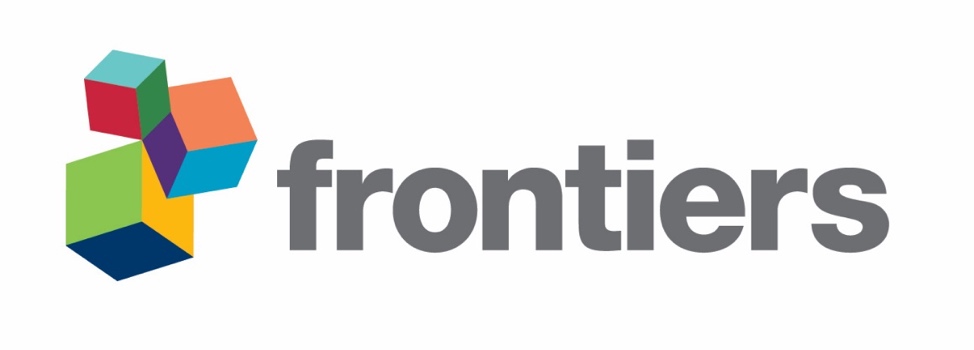
**

**
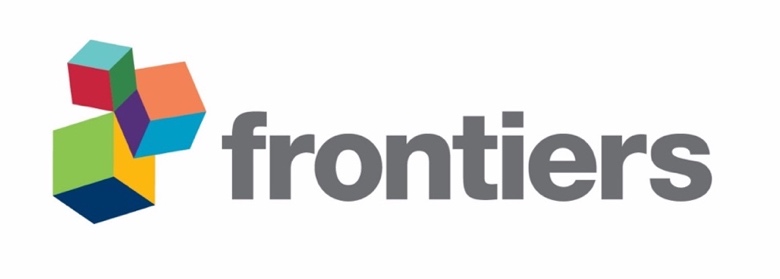
Supplementary Figure 1.** Flow diagram summarizing the identification, screening, eligibility assessment, and inclusion of studies in this narrative review. A total of 2,623 records were identified through database searches (PubMed). After duplicate removal, 2,582 records were screened, and 186 full-text articles were assessed for eligibility. Following full-text evaluation, 52 studies met the inclusion criteria and were included in the final narrative synthesis. The study selection process was managed using the Covidence systematic review software.
